# Secondary structure of nrDNA Internal Transcribed Spacers as a useful tool to align highly divergent species in phylogenetic studies

**DOI:** 10.1590/1678-4685-GMB-2016-0042

**Published:** 2017-02-13

**Authors:** Giovanna C. Giudicelli, Geraldo Mäder, Gustavo A. Silva-Arias, Priscilla M. Zamberlan, Sandro L. Bonatto, Loreta B. Freitas

**Affiliations:** 1 Laboratory of Molecular Evolution, Department of Genetics, Universidade Federal do Rio Grande do Sul (UFRGS), Porto Alegre, Brazil; 2 Laboratory of Genomic and Molecular Biology, Pontifícia Universidade Católica do Rio Grande do Sul, Porto Alegre, RS, Brazil

**Keywords:** Passiflora, lowest energy state, hairpins, ITS1, ITS2

## Abstract

Recently, it has been suggested that internal transcribed spacer (ITS) sequences are under selective constraints to preserve their secondary structure. Here, we investigate the patterns of the ITS nucleotide and secondary structure conservation across the *Passiflora* L. genus to evaluate the potential use of secondary structure data as a helpful tool for the alignment in taxonomically complex genera. Considering the frequent use of ITS, this study also presents a perspective on future analyses in other plant groups. The ITS1 and ITS2 sequences presented significant differences for mean values of the lowest energy state (LES) and for number of hairpins in different *Passiflora* subgenera. Statistical analyses for the subgenera separately support significant differences between the LES values and the total number of secondary structures for ITS. In order to evaluate whether the LES values of ITS secondary structures were related to selective constraints, we compared these results among 120 ITS sequences from *Passiflora* species and 120 randomly generated sequences. These analyses indicated that *Passiflora* ITS sequences present characteristics of a region under selective constraint to maintain the secondary structure showing to be a promising tool to improve the alignments and identify sites with non-neutral substitutions or those correlated evolutionary steps.

## Introduction

Ribosomal DNA (rDNA) consists of one of the largest multigenic families in eukaryotic genomes and is present at one or several locations in arrays of tandem elements (Hillis and Dixon, 1991). Each unit is composed of three rRNA gene regions (18S, 5.8S, and 26S) that are preceded by an external transcribed spacer, and the two internal transcribed spacers ITS1 and ITS2 separate the genes respectively (Buckler-IV *et al.*, 1997). The rDNA exons are highly conserved across eukaryotic organisms, whereas the ITS regions present length variability due to point mutations and insertions/deletions (indels) (Baldwin *et al.*, 1995; Álvarez and Wendel, 2003). Gene regions are further processed to produce the mature RNAs to form the cytoplasmic ribosomes, whereas ITS regions suffer a specific cleavage during the maturation of the ribosomal subunits and are thus not incorporated into mature ribosomes (Hillis and Dixon, 1991; Baldwin *et al.*, 1995). ITS regions play an important role in rRNA processing. Deletion of the central portion of ITS1, including a processing region that is used in the early stages of ribosomal maturation, blocks the formation of 40S subunits (Musters *et al.*, 1990), whereas small deletions in the 5'-terminal region of ITS2 prevent the maturation of 26S rRNA, and deletions in the 3'-terminal portion severely reduce the efficiency of this process (van der Sande *et al.*, 1992). The maturation and splicing processes depend on the secondary structure of ITS regions and thus imply some degree of conservation at the sequence or structural level (Mai and Coleman, 1997).

ITS sequences have been widely used in the inference of phylogenetic hypotheses or in molecular evolution studies in plants (Álvarez and Wendel, 2003), including several taxonomic levels (Kress *et al.*, 2002; Zimmer *et al.*, 2002; Koch *et al.*, 2003; Rauscher *et al.*, 2004; Bellarosa *et al.*, 2005; Kan *et al.*, 2007; Queiroz *et al.*, 2011; Ghada *et al.*, 2013; Fan *et al.*, 2014). In the *Passiflora* genus, ITS regions are the most commonly used marker for estimating the phylogenetic relationships among subgenera and species (Muschner *et al.*, 2003; Krosnick and Freudenstein, 2005; Lorenz-Lemke *et al.*, 2005; Koehler-Santos *et al.*, 2006; Mäder *et al.*, 2010; Cazé *et al.*, 2013; Krosnick *et al.*, 2013; Ramaiya *et al.*, 2014), although this is usually combined with other sequences in these studies.

The *Passiflora* genus is the largest member of the Passifloraceae family, with more than 520 species widely distributed in the Neotropical region and a few species occurring in the Old World (Deginani, 2001; Ulmer and MacDougal, 2004). This genus was initially divided into 22 or 23 subgenera based on floral morphology (Killip, 1938; Escobar, 1989), but the current infrageneric classification (Feuillet and MacDougal, 2003) regrouped the species into four subgenera that have been partially or fully corroborated in phylogenetic studies (Muschner *et al.*, 2003, 2012; Yockteng and Nadot 2004a; Hansen *et al.*, 2006, 2007): *Astrophea* (DC.) Mast., *Decaloba* (DC.) Rchb., *Deidamioides* (Harms) Killip, and *Passiflora*. The species of the *Passiflora* genus present a large diversity of floral and vegetative features, which contributes to the complex taxonomy of this group (Krosnick and Freudenstein, 2005).

The first study to obtain a molecular phylogeny for *Passiflora* (Muschner *et al.*, 2003) described a high divergence among ITS sequences of the different subgenera; consequently, alignment of these sequences presents several ambiguous regions and indels. For that reason, authors suggested a separate alignment per subgenus. Several plastid regions (Muschner *et al.*, 2003; Krosnick and Freudenstein, 2005; Hansen *et al.*, 2006) and other nuclear markers (Yockteng and Nadot, 2004a,b; Muschner *et al.*, 2012) have also been used in *Passiflora* studies; however, they do not present sufficient variability to distinguish species as efficiently as observed in ITS. Despite this, the low variability in plastid and other nuclear regions is advantageous in relation to ITS because allows the alignment of all subgenera simultaneously (Muschner *et al.*, 2003; Mäder *et al.*, 2010).

Mutations in the tandem repeats of rDNA are commonly homogenized through concerted evolution (Arnheim *et al.*, 1980; Baldwin *et al.*, 1995). This phenomenon makes each copy of an rRNA array very similar to other copies within individuals and species, thus providing a high degree of similarity in gene family and affecting the region variability (Hillis and Dixon, 1991; Buckler-IV *et al.*, 1997). However, the high level of ITS polymorphism present in *Passiflora* species indicates that the process of concerted evolution is not sufficiently fast to homogenize the region (Lorenz-Lemke *et al.*, 2005; Koehler-Santos *et al.*, 2006), as observed in other plant groups (Wissemann and Ritz*,* 2005; Harpke and Peterson, 2006). Melo and Guerra (2003) suggested that the high variability of ITS present in *Passiflora* could be related to the chromosomal location of rDNA in this genus, as has been suggested for *Gossypium* L. (Wendel *et al.*, 1995), because the 45S sites where the ITS region is located were mapped in subterminal position, which interferes with copy homogenization (Li and Zhang, 2002).

The presence of a high number of polymorphisms in sequences makes it more difficult to obtain good alignments, and this could affect phylogenetic inference (Álvarez and Wendel, 2003). For that reason, the aim of this study was to investigate the patterns of ITS nucleotide and secondary structure conservation across the *Passiflora* genus to evaluate the potential use of secondary structure data as a helpful tool to improve the alignments in a genus that presents high taxonomic complexity. We also evaluated for the first time whether the ITS sequences are under selective constraints, considering their secondary structure in *Passiflora* species. Considering the frequent use of ITS sequences, this study also presents a perspective for future analyses of secondary structure and estimates of phylogenies in other plant groups. Moreover, this original contribution improves the database of ITS secondary structure that is essential for a trustworthy reference to phylogenetic and DNA barcoding analyses (Ankenbrand *et al.*, 2015).

## Material and Methods

### Taxon sampling

GenBank sampling included 870 accessions of ITS1 and ITS2 from 163 species representatives of all four subgenera: *Astrophea* (9 spp.), *Decaloba* (112 spp.), *Deidamioides* (6 spp.), and *Passiflora* (36 spp.). We also included 74 new sequences (GenBank accessions numbers: KP769869- KP769905; KP769917- KP769953) from 26 species (*Astrophea* 5 spp., *Decaloba* 1 spp., and *Passiflora* 20 spp.), totaling 944 sequences from 189 *Passiflora* species. All these sequences were used to assess diversity in *Passiflora*. Subgenera were represented by different number of sequences according the number of species included on each one. Sequences that presented a large number of missing data in initial or final portions of ITS1 or ITS2 were not included in our analyses.

### Plant material, DNA extraction, PCR amplification and sequencing

To obtain the new sequences, the total DNA was extracted from young leaves dried in silica gel using the method of Roy *et al.* (1992). Voucher specimens were deposited at ICN Herbarium (Department of Botany, Federal University of Rio Grande do Sul, Porto Alegre, Brazil). ITS1 and ITS2 regions were amplified using primers 92 and 75 (White *et al.*, 1990) and amplification conditions as previously described (Desfeux and Lejeune, 1996). To exclude the presence of low stability templates, we used 10% dimethyl sulfoxide (DMSO) (Buckler-IV *et al.*, 1997; Fuertes-Aguilar *et al.*, 1999). We checked the quality and quantity of PCR products by horizontal electrophoresis in 1% agarose gel stained with GelRed (Biotium, UK), and purified them using the polyethyleneglycol (PEG) precipitation method (Dunn and Blattner, 1987). We performed the sequencing reactions automatically in a MegaBACE 1000 DNA Analysis System (GE Healthcare Biosciences, Pittsburgh, PA, USA).

### Data analyses

We removed the 5.8S gene region from ITS sequences because of its well-conserved nature, and ITS1 and ITS2 were analyzed individually. We discarded identical sequences obtained from a same species, such that each sequence type was considered only once, using DnaSP 5 (Librado and Rojas, 2009). Because *Passiflora* subgenera present high genetic variability among them, we conducted the analysis considering each subgenus separately. Consequently, for each subgenus, sequences of ITS1 or ITS2 were automatically aligned using default parameters in ClustalX (Thompson *et al.*, 1997), visually reviewed, and manually adjusted using MEGA6 (Tamura *et al.*, 2013). We deleted ambiguous sites from ITS sequences (Mäder *et al.*, 2010), and gaps were coded as binary characters (Simmons and Ochoterena 2000) using GapCoder (Young and Healy, 2003).

We selected 120 sequences from 60 species (*Astrophea* 6 ssp., *Decaloba* 39 ssp., *Deidamioides* 6 ssp., and *Passiflora* 9 ssp.) to model secondary structures of both ITS1 and ITS2 regions. For this selection, we first constructed phylogenetic trees in BEAST 1.8 (Drummond *et al.*, 2012) using the HKY substitution model with four gamma categories, a Yule tree prior, and 10^7^ chain lengths. The first 1000 trees were discarded as Òburn inÓ. We used the JModelTest software (Guindon and Gascuel, 2003; Darriba *et al.*, 2012) to select the best evolution model for our BEAST analysis, but because the resulting models were not available in the BEAST software, we selected the HKY substitution model as the closest to our results, based on *Passiflora* phylogenies previously obtained (Muschner *et al.*, 2003; Lorenz-Lemke *et al.*, 2005; Koehler-Santos *et al.*, 2006; Mäder *et al.*, 2010; Cazé *et al.*, 2013). Phylogenetic trees obtained using BEAST are available in Supplementary Material (Figures S1-S8). We selected the species to obtain the ITS1 and ITS2 secondary structures considering only species from well-supported clades (posterior probability 3 0.7). We included a greater number of *Decaloba* species to test differences between secondary structures of species from different supersections that are phylogenetically well supported (Krosnick *et al.*, 2013). For this analysis, we randomly chose three species from each supersection, except for *Multiflora*: all sequences available for this supersection were analyzed because it is the only paraphyletic supersection in *Decaloba* subgenus studies (Krosnick *et al.*, 2013).

We calculate mean guanine-cytosine (GC) content for each *Passiflora* subgenus and ITS segment (ITS1 or ITS2) separately. Considering these values and the size variation (bp) of the original ITS sequences, we generated 120 random sequences to evaluate whether the total number of secondary structures and lowest energy state (LES) values of ITS1 and ITS2 sequences were related to selective constraints. These sequences were obtained using a Random DNA Sequence Generator (www.faculty.ucr.edu/~mmaduro/random.htm) and presented the same length range (176 bp to 280 bp) of the original sequences. Therefore, for each *Passiflora* ITS1 or ITS2 sequence, we obtained a random sequence that presented the same size (bp) and a similar GC content. All secondary structures for those random sequences were also modeled, and the obtained information is available in Supplementary Material Table S2.

### Secondary structure prediction

We modeled ITS1 and ITS2 putative secondary structures for the 120 sequences of 60 *Passiflora* species and 120 random sequences using RNAstructure 5.3 (Reuster and Mathew, 2010). Output parameters of this software included the LES and total number of structures for each analyzed sequence. We manually analyzed the number of hairpins and paired nucleotides of ITS sequences as performed by Edger *et al.* (2014). Our analysis included for each *Passiflora* subgenus and random sequences: list of species, GenBank accession numbers, lengths, LES, total number of structures, number of hairpins, and number and percentage of paired nucleotides (Supplementary Material Tables S1 and S2).

We use the Wilcoxon-Mann-Whitney test in R software package (R Development Core Team, 2011) to assess whether there are significant differences between secondary structure parameters (i.e., LES, total number of structures, and number of hairpins) obtained for ITS1, ITS2, and random sequences. We performed an analysis comparing all ITS1 and ITS2 sequences to the 120 random sequences generated and another two analyses considering only ITS1 or ITS2 sequences and their 60 random sequences separately. We assessed differences for the same secondary structure parameters among the four *Passiflora* subgenera using a Kruskal-Wallis test (Kruskal and Wallis, 1952).

## Results and Discussion

### ITS1 and ITS2 sequences characteristics and secondary structure

Alignments for all subgenera presented indels with different extensions among species, as previously observed in other *Passiflora* studies (Muschner *et al.*, 2003; Mäder *et al.*, 2010). Characteristics of ITS1 and ITS2 regions evaluated per subgenera are summarized in Table 1. ITS1 sequences always presented higher length than the ITS2 sequences within each subgenus. *Decaloba* showed the longest length for ITS1 (336 base pairs - bp), as did *Passiflora* for ITS2 (239 bp). *Decaloba* exhibited the highest percentages of variable and informative sites for both ITS1 and ITS2 sequences, whereas *Deidamioides* showed the lowest percentages of variable and informative sites for ITS1 and *Astrophea* for ITS2 sequences.

**Table 1:** Characteristics of ITS1 and ITS2 dataset presented per *Passiflora* subgenera.

Subgenus	ITS region	N individuals	N species	Alignment length (bp)	Variable characters (%)	PI characters (%)
*Astrophea*	ITS1	26	14	288	31.25	20.14
	ITS2	31	14	235	28.51	16.17
*Decaloba*	ITS1	202	113	336	71.43	58.93
	ITS2	180	113	231	66.23	53.25
*Deidamioides*	ITS1	70	6	288	27.43	18.06
	ITS2	59	6	226	36.28	23.01
*Passiflora*	ITS1	126	56	260	46.54	31.54
	ITS2	106	56	239	46.86	25.94

ITS, internal transcribed spacer; BP, base pairs; PI, parsimony informative

Considering ITS1 and ITS2 sequences of all subgenera, the ITS1 region size range was between 220 and 280 bp, whereas ITS2 ranged between 176 and 222 bp. We evaluated each ITS1 and ITS2 sequence based on different parameters: sequence length, LES, total number of structures, number of hairpins, and number and percentage of paired nucleotides (Table 2; Supplementary Material Table S1). ITS1 sequences presented higher average length (mean 263 bp, Standard Deviation SD = 19 bp) than ITS2 sequences (mean 206 bp, SD 7 bp), considering the four subgenera. Additionally, ITS1 sequences displayed significant differences and lower mean values of LES than ITS2 sequences (-105.6 and −80.5 degrees, respectively; *P* < 0.001; Table 2). This result means that although they are of shorter sequence length, ITS2 sequences require more energy than ITS1 to form the secondary structures.

**Table 2:** Mean parameters values analyzed for ITS1 and ITS2 sequences per *Passiflora* subgenera. Standard deviations for each value are shown in parentheses.

ITS 1					
	*Astrophea* [6]*	*Decaloba* [39]*	*Deidamioides* [6]*	*Passiflora* [9]*	All sequences [60]*
Length (bp)	270.2 (2.3)	271.8 (8.8)	251.2 (19.7)	225.8 (2.5)	262.7 (19.1)
Lowest Energy State (LES)	-121.8 (5.5)	-104.2 (9.4)	-104.4 (4.9)	-101.8 (6.1)	-105.6 (9.8)
Total number of Structures	15.8 (3.9)	12.6 (4.1)	16 (4.5)	8.8 (3.8)	12.7 (4.5)
No. Hairpins	5.8 (1.6)	5.6 (1.4)	5.2 (2.1)	4.0 (0.9)	5.3 (1.5)
No. Paired Nucleotides	168.3 (2.7)	167.7 (10.8)	149.3 (12.6)	135.3 (7.3)	161.1 (15.7)
% Paired Nucleotides	62.3 (0.8)	61.7 (2.8)	59.4 (0.5)	59.9 (3.1)	61.3 (2.7)

ITS 2					
Length (bp)	208.8 (7.3)	208.1 (3.2)	204.5 (5)	195.3 (10.9)	205.9 (7.1)
Lowest Energy State (LES)	-90.3 (1.5)	-74.9 (6.2)	-87.9 (7.9)	-92.9 (6.1)	-80.4 (9.7)
Total number of Structures	14.2 (2.2)	11.2 (4)	13.8 (3.6)	17.3 (3.8)	12.7 (4.3)
No. Hairpins	2.8 (1.3)	3.3 (0.6)	2.3 (0.8)	2.8 (1)	3.0 (0.8)
No. Paired Nucleotides	128.7 (4.5)	123.4 (6.1)	128.7 (4.1)	130.7 (10.5)	125.5 (7.1)
% Paired Nucleotides	61.6 (1)	59.3 (3)	62.9 (2.6)	66.8 (2.6)	61.0 (3.8)

* Numbers of analyzed sequences. ITS, internal transcribed spacer; BP, base pairs

ITS1 sequences exhibited higher values of variable and informative characters, whereas the ITS2 region showed higher numbers of conserved sites, as previously observed for a different *Passiflora* species set (Giudicelli *et al.*, 2015). High conservative patterns in ITS2 sequences have been previously reported and related to structural constraints that are present at very deep phylogenetic scales in eukaryotes (Mai and Coleman 1997; Shultz *et al.*, 2005). Although highly conserved motifs have also been reported for ITS1 sequences in plants, such motifs seem to be shorter than those observed in ITS2, and in *Passiflora* ITS1 region, remaining parts of the sequence are more variable these motifs (Liu and Schardl 1994).

**Figure 1:**
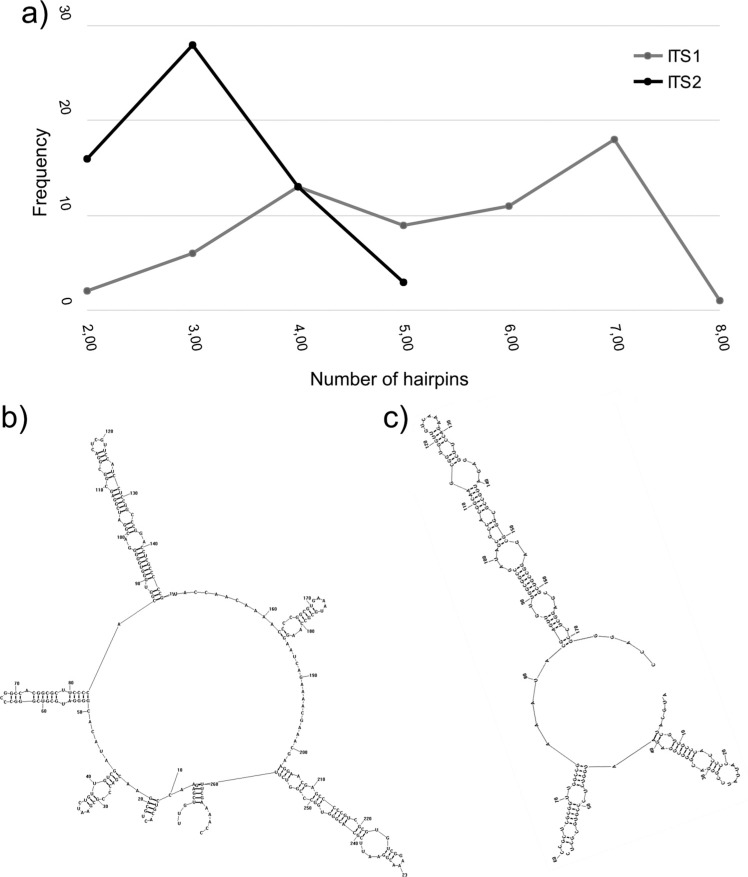
Frequency and examples of number of hairpins for ITS1 and ITS2 sequences. (a) Frequency of the total number of hairpins for ITS1 (gray) and ITS2 (black) sequences. Secondary structures examples with (b) seven hairpins in ITS1 and (c) three hairpins in ITS2.

No statistically significant differences were observed between ITS1 and ITS2 in the total number of possible secondary structures. ITS1 and ITS2 sequences exhibited statistically significant differences in the numbers of hairpins (*P* < 0.001; Table 2; Figure 1). The most frequent number of hairpins for ITS1 was seven (min = 2, max = 8), whereas the most frequent number of hairpins in ITS2 was three (min = 2, max = 5). No differences were observed in the percentage of paired nucleotides for ITS1 and ITS2 sequences (Table 2). Sequences had over 50% of their bases paired with other nucleotides to form secondary structures (Table 2), as observed for Brassicaceae (Edger *et al.*, 2014). Because ITS1 sequences displayed longer lengths than ITS2 and because the sequences exhibited significant differences according to the number of paired nucleotides, ITS1 presented a higher number of paired nucleotides (*P* < 0.001; Table 2).

Statistical analyses conducted for the four subgenera separately support significant differences between LES values for ITS1 (*P* < 0.01) and ITS2 (*P* < 0.001) sequences. These differences are not surprising when taking into account the large observed differences of the sequences among the four *Passiflora* subgenera. The highest mean value of LES for ITS1 was observed in *Passiflora* subgenus (-101.8 degrees), and for ITS2 in *Decaloba* (-74.9 degrees; Table 2). There were also statistically significant differences in the total number of possible secondary structures among subgenera for ITS1 (*P* < 0.01) and ITS2 (*P* < 0.01). For ITS1, *Deidamioides* showed a higher mean value of possible alternate secondary structures (16 possible structures, Table 2), while for ITS2 this was the case for *Passiflora* (17 possible structures; Table 2). No statistically significant differences were observed within *Decaloba* when the species were separated according to the supersections previously proposed (Feuillet and MacDougal, 2003; Ulmer and MacDougal, 2004). We also observed that different sequences types of a species did not exhibit differences on their secondary structure (data not shown).

### Comparison of secondary structures of ITS1, ITS2, and random sequences to assess selective constraints of ITS regions

**Figure 2:**
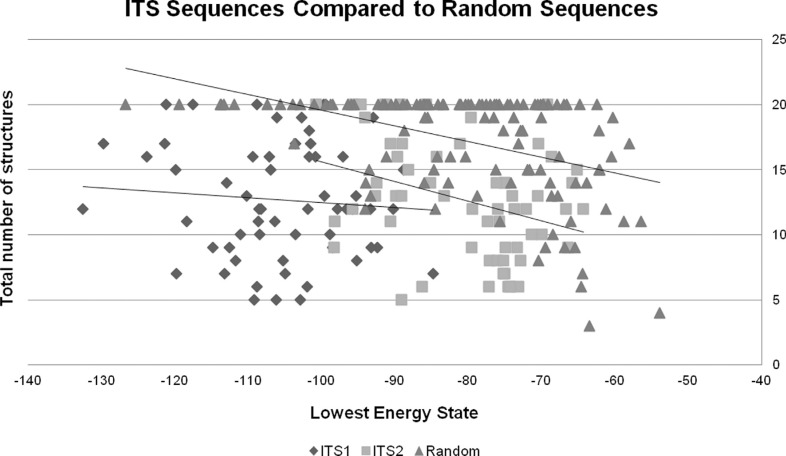
ITS sequences compared with random sequences. Scatter plot of t lowest energy state values (X-axis) and total number of secondary structures (Y-axis). Squares represent ITS1 sequences; circles represent ITS2 sequences; and triangles represent randomly generated sequences.

Randomly generated sequences presented −80.2 degrees for LES (min = −126.7, max = −53.9) on average and a mean of 17.2 possible secondary structures (min = 3, max = 20). In all three analyses comparing ITS and randomly-generated sequences (Figure 2), we found different values: to combined ITS sequences, LES *P* < 0.001 and number of possible secondary structures *P* < 0.001; ITS1 only, LES *P* < 0.001 and number of possible secondary structures *P* < 0.001; and ITS2 only, LES *P* < 0.001 and number of possible secondary structures *P* < 0.001. Those analyses indicated that *Passiflora* sequences secondary structures exhibit significantly lower energy states and less structures compared with randomly generated sequences that are similar in length and GC content. These results suggest that *Passiflora* ITS sequences show characteristics of a region that is under selective constraint to maintain its secondary structure.

Different secondary structure predictor softwares may propose very distinct structures from the same sequence, even when using the same initial parameters (Gottschling and Pltner, 2004). Additionally, thermodynamically optimal structures certainly do not reflect the structures in cells, because there are many unaccounted biochemical factors that give a dynamic (temporarily unstable) characteristic to the secondary structure of the rRNA transcripts. However, in addition to the strong differences seen among the *Passiflora* sequences, there are highly conserved structural patterns and sequence motifs for the entire genus, which support the suggestion of selective constraints for maintaining the secondary structures.

### Utility of ITS secondary structures to improve alignments

Analyses conducted for the four *Passiflora* subgenera separately revealed no statistically significant differences in the total number of hairpins among subgenera. However, although no pattern was observed to characterize each subgenus, secondary structures were shown to be a potential tool for improving the quality of alignments and identifying possible sites with non-neutral substitution patterns or sites with correlated evolution. This allows to obtain better results in phylogenetic inferences for *Passiflora,* as previously observed for other plant groups (Gottschling *et al.*, 2001; Goertzen *et al.*, 2003).

The highly conserved motif in the central region of the ITS1 sequence previously described for flowering plants (Liu and Schardl, 1994) was also present in the *Passiflora* sequences and was helpful in aligning the *Passiflora* sequences for phylogenetic analysis. Other ITS1 conserved motifs described in other groups were also found in *Passiflora*, such as 5'AAGGAA 3' in the central region of ITS1 (Liu and Schardl, 1994; Gottschling *et al.*, 2001) and a region rich in adenine and cytosine (Coleman *et al.*, 1998). Conserved patterns that had already been described in angiosperms (Hershkovitz and Zimmer 1996; Mai and Coleman, 1997) and green algae (Mai and Coleman, 1997) were also observed in *Passiflora* ITS2 sequences. These conserved motifs are commonly associated with the formation of hairpins in secondary structure.

Comparisons among species from different subgenera that presented the same number of hairpins showed conserved patterns on secondary structure that could be useful for improving the *Passiflora* sequence alignments. For example, when comparing the ITS1 region among species from the *Astrophea*, *Decaloba,* and *Deidamioides* subgenera with seven hairpins, we observed a common hairpin with 5-6 paired bases formed by initial (5'-TCGAA) and final (TTCGA-3') portions. This same motif was also found in ITS1 sequences that presented different numbers of hairpins, thus demonstrating the conserved nature of ITS1 sequences and serving as a reason why a universal set of primers could be used to amplify sequences of plants and fungi (White *et al.*, 1990; Álvarez and Wendel, 2003). The analysis of secondary structure also facilitates the identification of indels when sequences with different number of hairpins are compared, thus contributing to the improvement of the alignment of sequences with different lengths and high diversity index.

The conservation of specific nucleotides in secondary structures contributes to positional homology and is a helpful tool to improve the alignments (Keller *et al.*, 2010). Therefore, we believe that analyses of ITS secondary structures obtained from the *Passiflora* species could be a valuable source of information to improve alignment, considering the difficulties of aligning species from the different subgenera (Muschner *et al.*, 2003; Mäder *et al.*, 2010), and the same strategy could be used in other plant genera that present a complex taxonomy.

Many *Passiflora* studies have used ITS sequences to estimate phylogenetic relationships, and our results show that ITS regions are under selective constraints to maintain their secondary structures, likely due to their specific functions in the splicing process, as was observed for Brassicaceae (Edger *et al.*, 2014). The use of ITS as a molecular marker carries some risk and must be done cautiously, but it presents the advantage of having a great deal of sequence data for this locus (Krosnick *et al.*, 2013). Plastid markers are not sufficiently variable in the *Passiflora* genus (Muschner *et al.*, 2003; Mäder *et al.*, 2010; Cazé *et al.*, 2013), and ITS secondary structures could thus be a helpful tool to improve phylogenetic inferences and evolutionary studies in this genus and others.

## Supplementary material

The following online material is available for this article:

Figure S1 - Phylogenetic trees based on ITS sequences for *Astrophea*, ITS1.

Figure S2 - Phylogenetic trees based on ITS sequences for *Astrophea*, ITS2.

Figure S3 - Phylogenetic trees based on ITS sequences for *Decaloba*, ITS1.

Figure S4 - Phylogenetic trees based on ITS sequences for *Decaloba*, ITS2.

Figure S5 - Phylogenetic trees based on ITS sequences for *Deidamioides*, ITS1.

Figure S6 - Phylogenetic trees based on ITS sequences for *Deidamioides*, ITS2.

Figure S7 - Phylogenetic trees based on ITS sequences for *Passiflora*, ITS1.

Figure S8 - Phylogenetic trees based on ITS sequences for *Passiflora*, ITS2.

Table S1 - ITS1 and ITS2 sequences parameters analyzed

Table S2 - Random sequences parameters analyzed.
