# Supplementary figures and images for: Secondary structure of nrDNA Internal Transcribed Spacers as a useful tool to align highly divergent species in phylogenetic studies
Source: Genet Mol Biol. 2017 Feb 13;40(1 Suppl 1):191–9. doi: 10.1590/1678-4685-GMB-2016-0042 (PMC5452138; doi:10.1590/1678-4685-GMB-2016-0042)

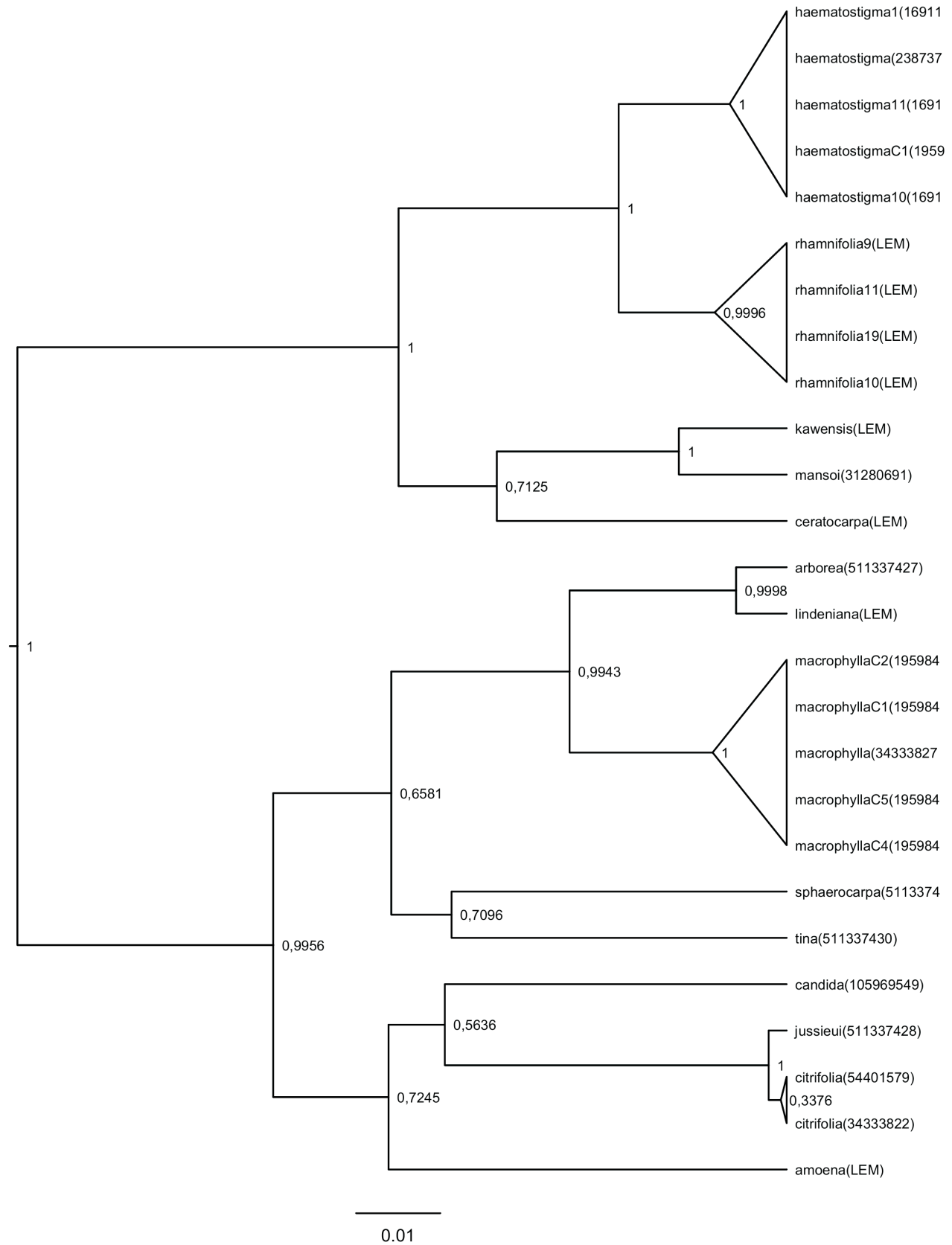

**Figure S1** - Phylogenetic trees based on ITS sequences for *Astrophea*, ITS1.

Supplement: Supplementary file 1 [file 1415-4757-gmb-1678-4685-GMB-2016-0042-Suppl01.pdf]

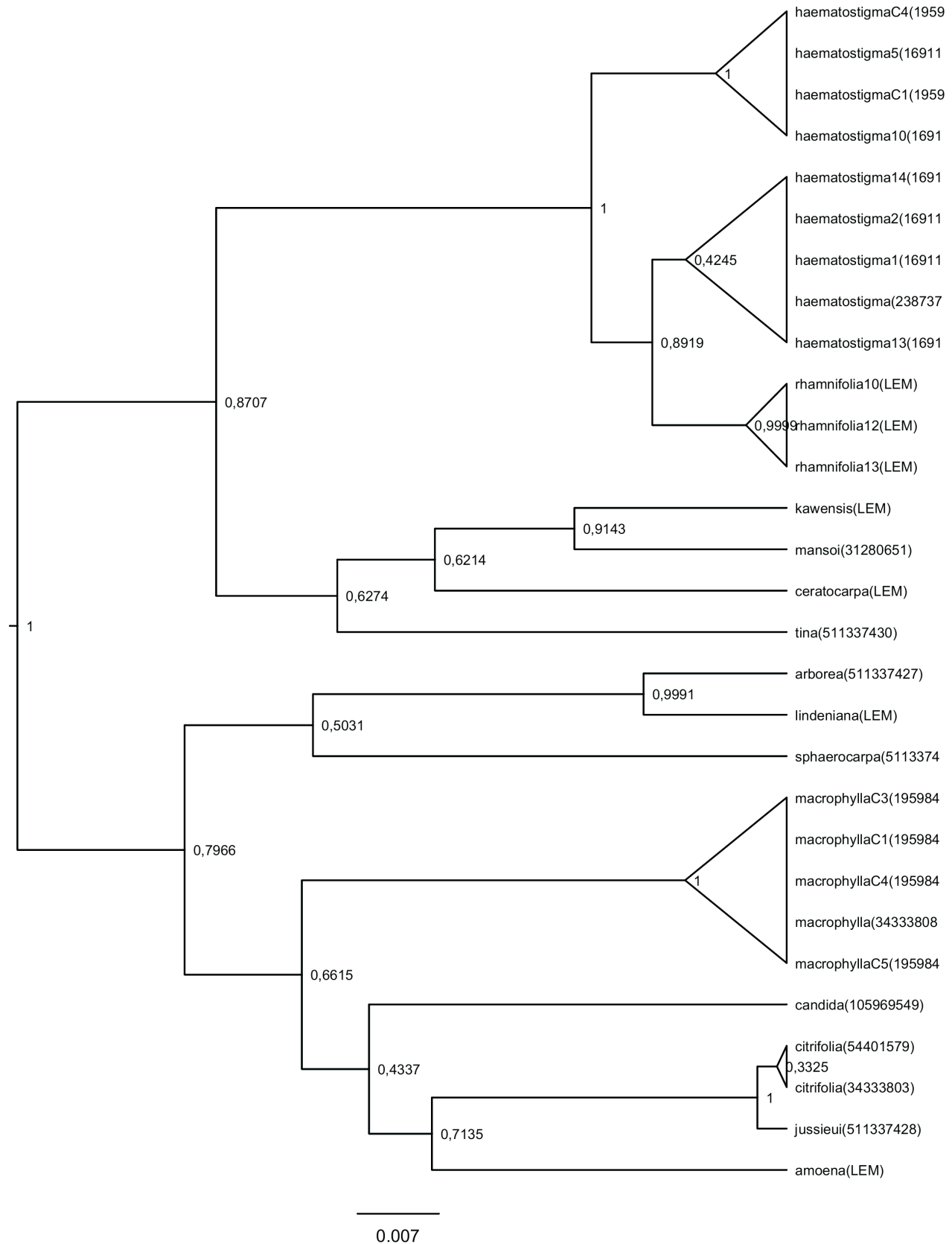

**Figure S2** - Phylogenetic trees based on ITS sequences for *Astrophea*, ITS2.

Supplement: Figure S2 [file 1415-4757-gmb-1678-4685-GMB-2016-0042-Suppl02.pdf]

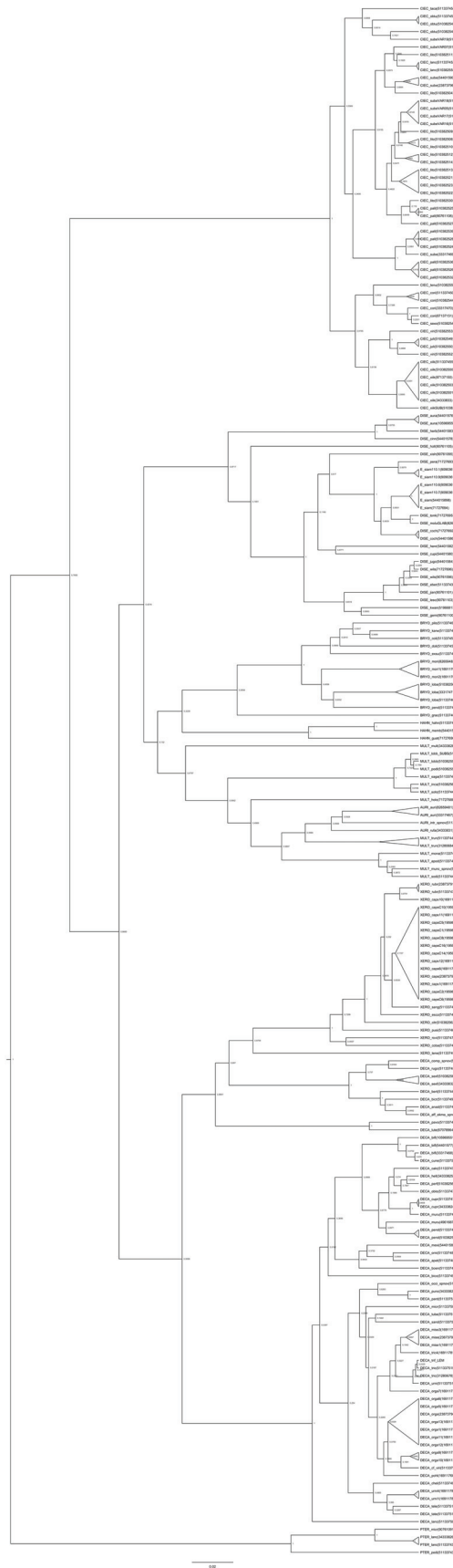

**Figure S3** - Phylogenetic trees based on ITS sequences for *Decaloba*, ITS1.

Supplement: Figure S3 [file 1415-4757-gmb-1678-4685-GMB-2016-0042-Suppl03.pdf]

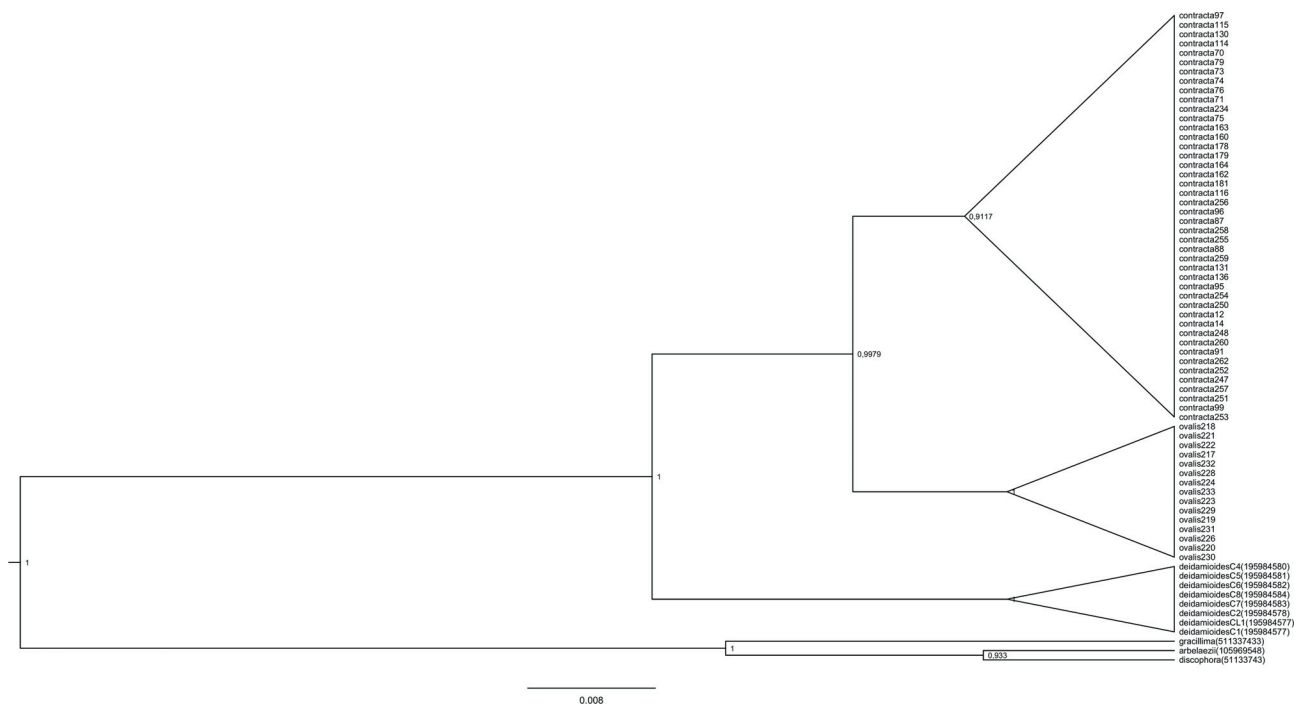

**Figure S5** - Phylogenetic trees based on ITS sequences for *Deidamioides*, ITS1.

Supplement: Figure S5 [file 1415-4757-gmb-1678-4685-GMB-2016-0042-Suppl05.pdf]

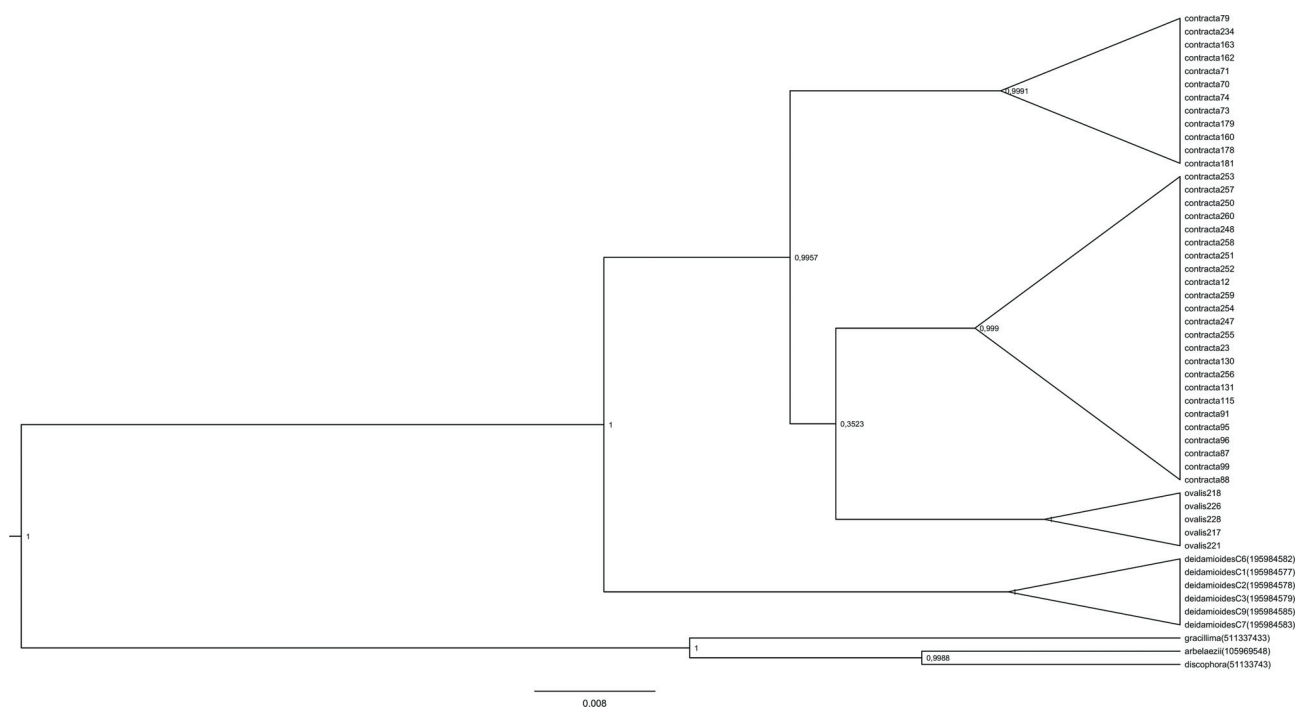

**Figure S6** - Phylogenetic trees based on ITS sequences for *Deidamioides*, ITS2.

Supplement: Figure S6 [file 1415-4757-gmb-1678-4685-GMB-2016-0042-Suppl06.pdf]
