# Supplementary material for: Secondary structure of nrDNA Internal Transcribed Spacers as a useful tool to align highly divergent species in phylogenetic studies
Source: Genet Mol Biol. 2017 Feb 13;40(1 Suppl 1):191–9. doi: 10.1590/1678-4685-GMB-2016-0042 (PMC5452138; doi:10.1590/1678-4685-GMB-2016-0042)
Supplement: Supplementary file 3 [file 1415-4757-gmb-1678-4685-GMB-2016-0042-Suppl10.pdf]

Table S2. Random sequences parameters analyzed: length, lowest energy state, total number of structures, number of hairpins, and percentage of paired bases.

Number	Random sequence	Lenght (176-280pb)	Lowest Energy State	Total Number of Structure	No. Hairpins	No. Paired Nucleotides	% No. Paired Nucleotides
1	CGTGTATTCGTTACGGCACCAGGGAGTTCGGGCCGAGTCAATGGAG<br>CTCGCAATGCGGAGTCTACCGCGTCCTGCCCCAACCGACGGGCTGT<br>GATCGACCACGAGCCAAGCCATCGCCTCTCGGGCACGCCGCTACAG<br>CGGTTACGAAGACCTTGCGGGGCATGGCCGCGGCCCGTTCAGCCAC<br>GTCCGAGGGCAGAGACCTGCCCCCATCCGTACGCTCGGCTATCTTCT<br>ACCCGTCCCCGGGGGCTAGGCAGGTCGTGGGGTGCGG	268	113,7	20	3	178	66,42
2	AGGAGGCCCTCGATCATCCCGTGGGACATCGACCTTCCCCTTGATAA<br>AGCGCCCCGCTCGGGTGTGGCAGAGAGGACGCCTTCTGAGTTGTGC<br>CATCCCTCGACCTCACCGAAGCTTGCTACCAATAATTAGGATCGCTG<br>CCTCGCGACAGACCTCCCGCCCACACCGCCCCGCGCTGAGCTAGCCA<br>GCGAGCGGTTAGCCTGACCCGCTCTCTAGGGTCGCGAGTACGTGAG<br>CCAGGGCTCCGGACCGGGCCATATAGTCGGGCCCGATCTC	273	95,6	20	5	156	57,14
3	GCCCCGACAACTGCAAGCCTCAGCTTCTTCGGGTAACGTGGTTAGCC<br>GAAGTTGCACGAGGTGCCGGCCGCGGACTGCTCCCCGGGTGTGGC<br>TCCTCCATCCGACAACGCGCGACCGCCATCGCCGCCGATTGGTTCGG<br>CGGACGGTGCCGTCGTCGTAGCTCGGGCATGTTTCCCTTGTGGGTG<br>TGAGGCCACCTGGCTTCGCGCCGTGGTCCCAAAGGAAAACCTATGG<br>ACTATGTTCCGGGTGGCACCGGGAATCTGAAGCGCGTGT	270	126,7	20	4	180	66,67
4	ATGTGGGCGTGGCGCGCGTGCGCCTTAATCTCCGCTCCATGCTAGG<br>GGTCTGGCTGCATGCTACGTTGACACACCCACGCCGCCCGGGGAAA<br>ATATGCGAGGCGGGCGGCCTGGCCGGAGCCCTACCGCGCCGGCAC<br>GTGTTCGTTTACTGTTGACTGGCGGCACATAAGCAATACCGTGGTCC<br>GTCAACCCCGGCCCTCTTACCCCCGGCGTTATGCGTCAAATGGCGCA<br>GGTCCGGATTGGCTGCGCGACGGTACCTGCTGGTCGGT	269	111,9	20	3	164	60,97
5	AGGGAGACCGAGAGTCCGTCGGGCCATGTCGCTAAAGCTTTCCGAA<br>CGCCCCGTGCCGACGCCGGGCGAGTCGGTGCACGCTCCCCTCCTTG<br>GAGGCGCGTAGACATACGAGCGGGCAGATGATGCGTACGCCCCCC	273	113,8	20	4	156	57,14

Number	Random sequence	Lenght (176-280pb)	Lowest Energy State	Total Number of Structure	No. Hairpins	No. Paired Nucleotides	% No. Paired Nucleotides
6	CGATGCGTCCAGCACCCCACGCCCCCTCCGAGAGCTGGAAGGGCAC<br>CCTGCACTTGGATGGGGGACTATCTCGTGGGGCGAGCCCGCACCGT<br>CACTCGTGCGGAAGAGTTAACACGGTTGGGAGCAGGGGTGGTTT<br>CGCCGGTGGCGGCGGCTAACCATGTCCCCTGCCACTGCAGCTGTAT<br>CTAAGCCGTGCAATGGGAACATCCACACCTCGGTGAACCGATGCGC<br>CGCTTCGGGGTACCGTTTTGGCTACCTGTTACTAAGCCCATCGCGGT<br>CCTCAGGCATCGCGCACGTAGGGCCGCACCGCGCGCATGTCGAACT<br>GGTGGCGAGGTACGATCCCACGACCGGCGTACGATCCAACTGCGCG<br>GGCGTGACGAGCTTCTTATATACGCTTCGCCCGCCGG	268	107,4	20	7	166	61,94
7	ACCGGCCTCGCGATGGGGCGGCCGCGCACGAGCTTATGACAAGTA<br>ACGAGCGTGTACTCGTTTAGCCACCTCGCAGTGAAAGCCGGGAGAA<br>TGGGGGCCGCTACACACACTTTACCGCAACTACGCCTAACTGAGATA<br>CTGCCATGGACGACTACCCATCCCTCTGGGCCTCAGACAGCCGGATA<br>CAGTGACTTTGATAGGTTT	204	56,5	11	3	108	52,94
8	GCGGGGCACGGCTGCGACCCGCACAGCCGCGTGCGGGGGGAGGA<br>ACCTTTGCGCGTTAGTATGTTGACCCGTGTACTACGCATGCGGGCAG<br>GTCATGTGGGTGGAGACATCCGGGCCAGGCTCTCGACCTTCCCGTG<br>GGAGGTGATCCAGCCCGCTGTAGGACCACCCCGCTCGGGCGTGGCA<br>CTGAGCACGCCGTCCCCATTCTGGTAACCGTCGTCCCTA	222	96,0	20	5	136	61,26
9	TCAGGGCTTGGAGCGAGTGGTGACGGTTATTCCCCAGGGACGGAC<br>CTCCTACTCACAGTCGGTCACATTGGGCTACTCCCTGGGCCTTCCGC<br>TTGGCCCGGTCTGTTGGGCCGCCACTGCGTGAGCTTCGGCCCCGCG<br>CTGCGCTGTGTCGGCGGCTCTCATCGGGGCCCCACATCTGGAAACC<br>CCGACCTATTTTGACGGCACCGTTGGCG	212	85,5	19	4	128	60,38
10	GGGTCCGTAGCGCGTCCATCGCGTCCCGGCGCGCGTTCAGCTTGAC<br>GACCCCTTGGCGCAAGGGTGCTGGCCGCGTGCTAGGTTGAGGCGG<br>CTGCACTGCTGCAAGGTCCGTTGCGGAGGGGGCGGCCTGGGGGA<br>GCGCTAGCCCGTCGGCCCGTGCAGGAACACTCTATATTGCTCCCGG<br>ACGGGCAGATTGCTAGAGCGCCGC	206	92,3	20	4	134	65,05
11	TTTCAGCCCCCCTGTCGCCGCCGACGTCTGTGATATGGCGTTGTTGC	207	69,1	20	4	124	59,90

Number	Random sequence	Lenght (176-280pb)	Lowest Energy State	Total Number of Structure	No. Hairpins	No. Paired Nucleotides	% No. Paired Nucleotides
	GGTTCCACTCTATCGGGGCACCAGCTGATGCGTAGGGAGACCCGGA<br>ATGAGCCGGCCTATGTCACTGAAACTGTGCAAACGCCCGGTGCCGT<br>TGGTGTAGGTCCCGACCGACGCCCGCCTCGTTGAGAACCCGCAATC<br>TTACAACTGGGGACATGATCCC						
12	TACGCCCGTCATCTACTCGCGTCCCTGTGGCCCCAGTCCATGTGGTG<br>GGAGGGCACCCTCCACAAGGTCTGGCGCCATGGTGGTATGGCGAG<br>CCCGTACCGTGGTAGACGCGGCACGGGTAGGACCATCGGTAGTAG<br>GGATAGTGCGGAAGCTCGCAGACCACTGCCTATAGGGGGTGCCTGC<br>CTCTACGAAGAGCGACTGC	202	93,3	13	4	132	65,35
13	CAGTATAACCCCACGAGGATCCGAAAAGGCGAACCGGCCCAGACG<br>ATCCGGAGGCACGGGCCTCAAAGCCGCGACACGACGGCTGTCGGC<br>CGGTAACAGTAACCCCGGAGTGAACTCCTATGGGGCTGGATAGAAC<br>AGCCCTGGTGGGCCCCATCAGCAACCCGAATACGTGGCTTTTCGGG<br>AGGCGGCCGGAGGGGCGATGTCTTCCACTATTCGAGGCCGTTCGTT<br>AATACTTGTTGCGTTCCTAGCCGCTATATTTGTCTCTTTGCCGAC	273	99,4	20	5	162	59,34
14	TAATGTGGACAAGCACACCATAGCCATTTGTCGGAGCGCCTCGGAA<br>TACGGTATGAGCAGGCGCCTCGTGAGGCCATTGCGAATACCAGGTG<br>TCCTGTAAGCAGCGAAGGCCCGTACGCGAGATAAACTGCTAGGGAA<br>CCGCGTGTCTACGACCGGTGGTGGATTTAATCTCGCCGACGTGTAG<br>ACATTCCAGGCAGTGCGTCTGCCGCCGGGCCCCTCTGGTGACTGGG<br>TAGTTGGACTTGCCCTTGGAAGACATAGCAAGACCCTGCCTCTCTA	276	102,9	20	5	182	65,94
15	TTGATGTCACGGCGAATGTCGGGGAGACAGCAGCGGCTGCAGACA<br>TCAGACCGGAGCAACACTAACGTGGGATAACTCCGTAACTGACTAC<br>GGCCTCCTCTAGACCTTACTTGACCAGATACGCTGTCTTTGGCACGT<br>GGATGGTTTAGAGGAATCACATCCAAGACTGGCTAAGCACGAAGCA<br>ACTCTTGAGTGTAAAATTGTTGTCCCCTGTATTCGGGATGCGGGTAC<br>TAGATGACTGCAGGGACTCCGACGTCAAGTACATTACCCC	271	94,0	14	4	180	66,42
16	GTCATAGGCGCCGTTCAGGATCACGTTACCGCCATAAGATGGGAGC<br>ATGACTTCTTCTCCGCTGCGCCCACGCCAGTAGTGATTACTCCTATG<br>ACCCTTCTGAGAGTCCGGAGGCGGAAATCCGCCACGAATGAGAATG	273	105,6	20	5	162	59,34

Number	Random sequence	Lenght (176-280pb)	Lowest Energy State	Total Number of Structure	No. Hairpins	No. Paired Nucleotides	% No. Paired Nucleotides
17	TATTTCCCCGACAATCATTATGGGGCGCTCCTAAGCTTTTCCACTCGG TTGGGCCGGCTAGGCCTCCCTGCCCGGAGTTTCGGCGGACTGCTGC CGACACCCGGGCATTGTCTTAGGGGGGTTATTCGAGGGCA CCCGCAGCCAACTTGTCGGGACCAGCCGGGCTGGTCATCGGGCTTA TACAGCGAAATGCCGAGGACCCGGCCCCGCGCTATGGAACGTCTTT AGCTCCGGCAGGCAATTAAGGACAACGCAAGCATGGCGGATATAA ACAGAGAAACGGGCGAATACACCTGTTCGTGTCGTATCGGTAAATA GCCTCGCGGAGGCATGTGCCATGCTGGCCTGCGGAGCACTCTGGTT ATGCATATGGTCCACAGGACACTCGTCGCTTCCGGGTTTGCCCTC	274	98,5	20	3	168	61,31
18	TATGTGACGGTCTTTAGGCGCACTTATGCTCAGCACCGTTTAAACCA GACCGACACCAGATCTGTAAGGTCCGCCACGCAGACGAGAGCGCAC GGAGACCACCGAGCGATCTACCTGATCGGCGACCATCTGTGTGGTA CTGGGGCCGAGAGGTAACTACGGTGCCGCTAACAGCCCCTCGGTCG TCGCTGACGTCTGTAGTCTAGCCTCATTATGATCGTACGCTATTCAG GGATTGACTGATACCGGAAGACATCTCAGTTGAAGTG	269	88,4	20	3	170	63,20
19	GTGTATGCGACAGAGACCGTGCACCTACCAAACCTCCTTAGTCTAAG TTCAGACCAATTGGTAGTTCGTCCAGAACTCAGATTTTATCACCAGA GGACGCACGCCCTACCTCCATGATCCACTGACGTCCCTGAGGCTGCA ATACATGCAACCAGGCAGTCTCCGCGGTAAGTCCTAGTGCAATGGG GCTTTTTTTCCCTGGTCCTCGAGAAGAGGGGACGCCGGTCCAGACA TCTCTAATGTGGTAATTGGGAGGACTCTTGGCCCTCCGCC	273	78,1	20	3	158	57,88
20	CTTAGGCGGTGCATACTCTTCCATAAACGGGCTGTTAGTTATGGGGT CCGAGGATTGAAAAAGGTGAGCGAACTCGGCCGAACCGGAGAGAC GGGCTTCAAAGCAGCCTGACCACGGTTGCGCGTCCGTATCAAGATC CTCCCAATAAGCCCCCGTCACCGTTGGTTGTAGAGCCCAGGACGGG CCGGCCAGATGCGCGACTATATCGCTTAGCGGCTCTTGGGCCGCGG TGCGTTACCTTGCAGGAATCGAGGCCGTCCGTTAATTCCCC	271	91,4	20	3	154	56,83
21	TTGCATACATATCGCGTTTTTTTGTCCCTTTACCCGCTCACTTAGAAA AGGGACAGATAGCTTCTTACCGGTGCGCCTCCGTACGCGGTACGAT CGCACGCCCCGTGAGAACGATAGGTAAACCTGGTGTCCTGTGAGCG	273	88,9	20	5	164	60,07

Number	Random sequence	Lenght (176-280pb)	Lowest Energy State	Total Number of Structure	No. Hairpins	No. Paired Nucleotides	% No. Paired Nucleotides
22	GCGAAAGCCTAAACGGGAAATACGCGGCCATAAGTCGGTGCGAAT<br>ACGGGTCGTAGCAATGTTGGTCTGGCTATGATCTACATATTACAGGC<br>GGTACGTCTGCTCTGGTCAGCCTCTAGTGGCTCGTAAGATA<br>GTGCAGCCGCTGGTGATCACTCGATGACCTCGGCTCCCCATTGCTGC<br>TACGGCGATTCTTGGAGAGCCAGCTGCGTCCGCTAATGTGAGGACA<br>GTGTAGTATTAGCAAGCGATAAGTCCCCAACTGGTTGTGGCCTATC<br>GAAGAGTGAACTTCATAACACATGCTGTCCCACGCACGTGGATGGT<br>TTGGACAAATCTGATTCGAGTCTGATCAACCTTCA	220	69,5	9	3	142	64,55
23	CACAGATCTAGAGTCGAAAGCAGTGATCTCCCGGGTGCGAGATAAA<br>AATACTAGGTAACTAGAGGGTCTGCGACGTTCTAAACGTTGGTCCG<br>TCTGAACCGCCATCCAGGATCACGTCGCCCCGAAAAAAAGATATCA<br>GGAACTCTCCTCCTCAGCAGTCTGGTCTATGGAAACTACAGGACTAA<br>CCTTCCTGGCAACCGGGGGCTGGGAATCCGTCACATGAGTCAAGGT<br>ATTTGCCCGATAATCTATACTCCAGGCATCTAACTTTTCC	271	66,6	20	4	148	54,61
24	CACTGCCTTAAGCGGGCCTGCCCTTTCTGCCTGTCGATCCATAGGAC<br>TCGTGCCAACGCGCAGGCTTAGTTCGAGGAGAAATATCCGGGGCCA<br>AAGACAACCAGCATCTCGCGTCTTGCCCAACCCCCCTACACGCTGTT<br>ATAGGGAATCAGCGGGAACCCGGTGCCAGGCGATGGAACGTCCTT<br>AACTCTGGCAGGGAATTAAAGGGAACGTATATACAACGCAAAGAA<br>GCTGGAAAATTGGCGAGGGAATCCTGTCTCTGTCTATCCAAGA	273	75,8	20	4	146	53,48
25	ATGGGCACGGGGTGGCAACCGTCGTGCTAGCGTGCGGGGTGCACT<br>TCGTAACCATTTGGGACACCGGACACTCGCTGTTTTCGAAATTACCC<br>TTTAAGCGCGGGTATTGAACCAGGCTTATGCCCAGGATCGTAGCAA<br>GCAGACTCAAACAAGATGTATTTTGCCCGCCTTGCAGACGAAACCA<br>GTCGGAGGTTACGGAGCATACTATCACGTGGGCGGCCACTGGTGA<br>GCTACTGCACCCCAGGGGCAACGTTGATGCCCCTAAGAAGCTCTGG<br>CTGGA	280	98,8	20	3	178	63,57
26	CGCAAGCCGTAACACCCGTGTCACTTCATAATCGTTTGCAATTCAGG<br>GCTTGACCTACACTGGATTGCCATTCTCTCAAAGTATTATGCAGGAC<br>GGCGTGCGCGTTCCATGTAAACCTGTCATAACTTACCTGAGACTAGT	271	74,7	20	3	168	61,99

Number	Random sequence	Lenght (176-280pb)	Lowest Energy State	Total Number of Structure	No. Hairpins	No. Paired Nucleotides	% No. Paired Nucleotides
27	TGGAAGTGTGGCTAGATCTTTGCTCACGCGTCTAGTCGGTCCACGTT<br>TGGTTTTTAAGATCCAATGATCTCCAAAACGCTGCAAGATTCCCAAC<br>CTGCTTTACTAAGCGCTGGGTCCTACTCCAGCGGGA<br>CTTTTTATCTAAAGACGATGAGAGGAGCACTCGTCAGACCACATAGC<br>TTTCATGTCCTGATCGGAAGGATCGTTGGCGCCCGACCCTCAGACTC<br>TGTAGTGAGTTCTATGTCCGAGCCATTGCATGCGAGATCGGTAGATT<br>GATAGGGGACACGGAATATCCCCGGACGCAATAGACGGACAGCTT<br>GGTATCCTGAGCGCAGTCGCGCGTCCGAACCCAGCTCTACTTTAGA<br>GGCCCCGGATTCTGGTGCCCGCAGGCCGCAGAACCGATTGGGGG	276	85,9	20	5	170	61,59
28	CATGTACAACAATATCTGTTAGTCACCTTTGGGACACGGTCTCCCAC<br>CTCACTGGAATTTAGTCCCTGCTATAATTAGCCTTCCTCATAAGTTGC<br>GTTACTTCGGCGTCCCAACCGCACCCTTACCACGAAGACAGGTTCGT<br>CCATTCCCATACTGCGGCGTTGGCAGGGGGTTCGCATGTCCCACGC<br>GAAACGTTGCTGAAGGCTCAGGTCTCTGAGCGACAAAAGCTTTAAA<br>CGCGAGTTCCCGCCCATAACTTGGTCCGAATGCGGGTTCTTGC	277	75,8	20	3	144	51,99
29	ATCGTTCCACTGAGTTTGTTTCATGTAGGACGGGCGCAAAGTGTGCT<br>TAGTTCAACCTCCAATACCTCGTATCATTGTGCACCTGCCGGTCACCA<br>GCCAACGATGTGCGGACGGCGTTGCAACTTCCAGGGCCTAATCTGA<br>CCGTCCTAGATACGGCACTGTGGGCAGTACGAGGTAATGGCAGACA<br>CCCAGTGCCGAACAACACCTGACCTAACGGTAAGAGAGTCACATAA<br>TGCCTCCGGCCGCGTGCCCAGGGTATATTTGGTCAGTATC	273	88,7	18	2	180	65,93
30	GAATGGACTGAGATGAACCTTTACACCGAAGCGGAAACGGGTGCG<br>TGGACTAGCCAGGAGCAAACGAAAAATCCTGGCCTGCTTGATGTCT<br>CGTGACGTTCTTAGAGATGGACGAAATGTTTCGCGACCTAGGAAAA<br>GGTCGCCCTACAAAATAGATTTGTGCTACTCTCCTCATAAGGAGTCC<br>GGTGTACCGAAAGAACAAGGCGAGCCTAGGTAGCAACCGCCGGCT	274	85,4	20	4	146	53,28
31	GCGGGGGTAAGGTATCACTCAAGAAGCAGGCTCGGTAACACACGG<br>TCTAGCTGACTGTCTATCGCCTAGGTCATATAGGGACCTTTGATATC<br>TGCGTGTCCAGCCTTAGGATTCACTTCAGCGCGCAGGCTTGGGTCG<br>AGATAAAATCACCAGTGCCCAAGACCAGGGGGGCTCGGCGCGTTG	276	84,1	20	4	178	64,49

Number	Random sequence	Lenght (176-280pb)	Lowest Energy State	Total Number of Structure	No. Hairpins	No. Paired Nucleotides	% No. Paired Nucleotides
32	GCTAATCCCGGTACATCTTGTTATGAATATTCAGTAGAAAGTCTGTG<br>TTAGAGGGACGAGTCACCATGTACCAAGGGCGATATTAATCGGTGG<br>GAGTATTCATCGTGGTGAAGACGCTGGGTTTACGTGGGAAAGGTG<br>CTTGTGTCCCAACAGGCTAGGGTATAATGCTGGAACCGTCCCCCAA<br>GCGTTCAGGGTGGGGTTTGCTACGACTTCCGAGTCCAATGTGTCCG<br>TGTTCACGATATATGCGCTCAAGGGCGAGAATTGGACCTGGCTTTC<br>GCGTTAGTACGTAGCATGGTGACACAAGCACAGTAGATCCTGCCCG<br>CGTATCCTATGTATCAAGTTAGTTCTTATGGAATATAATAACATGTG<br>GATGGCCAGTGGTCGGTTGTTACACGCCTGCCGCGACGCTGAAT	275	85,9	19	4	182	66,18
33	GACCCGGACTAGAGTGGCGAGATCTATGGCGTGTGACCCGTTATGC<br>TCCATTTCGGTCAGTGGGTCACTGCTAGTAGTCGATTGCATTGCCAT<br>TCTCCGAGTGATTTAGCGTGACGGCCGCAGGGGACCCATAAAATGC<br>AATCGTAGTCCACCTGACCGTACTTAGAAATGAGGGTCCCCCTTTGC<br>CCACGCACCTGTTCGCTCGTCGTTTGCTTTTAAGGACCGCACGAACC<br>ACAGAGCATAAGGAGAACCTCTAGCTCCTTTACAAAGT	271	86,0	14	4	152	56,09
34	GCTGGTTCCCTTTCCAGCGGGATGCCTTATCTAAACGCAATGACAGA<br>CGTATTCCTCAGGCCACATCGCTTCCTACTTCCGCTGGGATCCATCGT<br>TGGCGGCCGAAGCCGCCATTCCATAGTGAGTCCTTCGTCTGTGTCTT<br>TCTGTGCCAGATCGTCTGGCAGATTGCCGATCCAGTTTATCCCACGA<br>AACTATAGTCGTACAGGCCGAAATCTTAAGTCAAATCGCGCGACTA<br>GGCTCAGCTCTATTTTAGTGGTCATGGGTTCTGGTCCG	273	80,0	20	4	156	57,14
35	CCCGAGCGGCGCAACCGATTAGGACCATGTAGAACATTTGTTACAA<br>GTCTTCTTTTAAACACAATCTTCCTGCTCAGTGGCGCATGGTTATCGC<br>TGTTGCTAGCCAGCGTGGTAAGTAACAGCACCACTGCGAGCCTAAT<br>GCGCCCTTTCCACGAACACAGGGCTGTCCGATCCTATATTAGGACTC<br>CGCGATGGGGTTGGCAAGTCGCACCCTAAACGATGTTGAAGGCTCG<br>CGATGCACACGCTCTGGTACAATACATACGTGTTCCGGC	272	87,0	20	4	180	66,18
36	TGTTATCCTGCATCGGAACCTCAATCATGCATCGCACCAGCGTGTTC<br>GTGTCATCTAGGAGGGGCGCGTAGGATAAATAATTCAATTAAGATG<br>TCGTTATGCTAGTGTACGCCTACCCGTCACCGGCCATCTGTGTGCGG	272	77,6	20	3	162	59,56

Number	Random sequence	Lenght (176-280pb)	Lowest Energy State	Total Number of Structure	No. Hairpins	No. Paired Nucleotides	% No. Paired Nucleotides
37	ATGGGGCGACGAGTTACTGGCCCTGATTTCTCCGCTTCTAATACCGC ACACTGGGCAATACGAGCTCAAGCCAGTCTCGCAGTAACGCTCATC AGCTAACGAAAGAGTTAGAGGCTCGCTAATTCGCACTGT CGGGGTCCCTTGGGTGTTTTGCGCTAGCGTCAGGTAGGCTAGCATG TGTCTTTCCTCCCAGGGGTATGCGGCTGCGTGGTCAAATGTGCAGC ATACGTATTTGCTCGGCGTGCTTGGTCTCTCGTACTTCTCCTGGAGAT CAAGGAAATGTTTCTTGTCCAAGCGGACGGCGGCTCTACGGAATGG ATCTACGTTACTGCCTGCATAAGGAGAGCGGAGTTGCCAAGGACGA AGGCGACCCTAGGTTCTAACCGTCGACTTCGGCGGAAAG	271	96,4	20	4	162	59,78
38	GTTTCACTCAGGAAGCAGACACTGATTGACACGGTCTAGCAGAACG TTTGAGGACTAGGTCAAATTGAGTGGTTTAATATCGGCATGTCTGG GATTAGAATACAGTATAGTGCGCTGATCGGAGACGAATTAAAGACA CGAGTTCCCAAAACCAGGCGGGCTCGCCACGACGGCTAATCCTGGT AGTTTACGTGAACAATGTTCTGAAGAAAATTTGTGAAAGAAGGACC CGTCATCGCCTACAATTACCTACAACGGTCGGCCGCACCTTC	272	67,0	20	4	152	55,88
39	GATTGTCGTGGCCACCCTCGGATTACACGGCAGAGGTGGTTGTGTT CCGACAGGCCAGCATATTATCCTGAGGCGTTACCCCAATCGTTCTCC GTCGGATTTGCTACAGCCCCTGAACGCTACATGCACGAAACCAAGTT ATGTATGCACTGGGCCATCAATAGGACGTAGCCTTGTAGTTAGCAC GTAGCCCGGCCGCATTAGTACAGTAGAGCCTCCGCCGGCATCCTGT TTATTAAGTTATTTCTACAGCAAAACGATCATATGCAGATCCGC	276	70,9	20	3	166	60,14
40	AGTGCGCGGTAGAGACACGTCCACCCGGCTGCTCTGTGACAGGGAC TAAAGAGGCGATGATTATCGTGAGTGCCCCGTTATGGTCGTGTTCG GTCAGAGCGCCCTTGCGAGCAGTCGTATGCTTTCTCGAATTCCGTGC GGTTAAGCGTGACAGTCCCAGCGAACCCACAAAACGTGATGGCAGT CCATGCGATCATACGCAAGAAGGATGGTCCCCAGACACCGGCGCAC CAGTTTTCACGCCGAAAGCATAAACGAGGAGCACAAACGAGAGT	275	81,2	20	4	166	60,36
41	GCTTGAACTGGACCTGTAGTTCCTCTACGAAGAACACCTTGAGCTGT CGCGTTGTTGCGCTGCCTAGATGCAGTGTCGCACGTATCACTTTTGC CTCAACGGCTGCTGCTTTCGCTGTAACCCTAGACAGGCAACAGTAAG	272	70,2	20	3	156	57,35

Number	Random sequence	Lenght (176-280pb)	Lowest Energy State	Total Number of Structure	No. Hairpins	No. Paired Nucleotides	% No. Paired Nucleotides
	CGCCTTTTGTAGGCGAGAGCTCCGCCTGTGACTAACTGCGCCAAAAC<br>GTCTTCCAATCCCCTTATCCAGTTTAACTCACCGAATTCTTGCGATTC<br>AGACCCTAATATCACATCATTAGACGCCAATTGCCT						
42	CTGCCAAAATTCTGTCCACAAGCGTTTTGGTTCGCCCCAGTGAAGTT<br>GCCAATAACGACCACCAAATCCGCATGTTACGGGACTTCTTATTAAT<br>TCTTTTTTCGTGGGGAGCAGCGGATCTTAATGGATGGCGCCAGGTG<br>GTATGGAAGCTAATAGCGCGGGTGGGAGGGTAATCAGCCGTCTCC<br>ACCAACACAACGCTATCGGGTCATACTATAAGATTCCGCAATGCGGC<br>TACTTATAGGATGCCTTAACGGTATCCGCAACTTGCGATG	272	86,6	20	4	158	58,09
43	TGCCTGCTATGCTTAAATGCATACCTCGCCCAGTAGCTTTCCAATATG<br>GGAGCATCAATTGTAGATCGGGCCGGGATAATCATGTCGTCACGGA<br>ACTTGCCGTAAGAGTAATAATTCAAAAGAGATGTCGGTTTGCTGGTT<br>CACGTGAAGGTCCCTCGCGCCACCTCTAAGTAAGTGGGCGGTCGTG<br>ACATTATCCCTGATTTTCTCACTACTATTAGTACTCACGGCGCAATTC<br>CACCACAGCCTTGTCTCGCCAGAATGCCGGTCAGCA	271	71,9	15	3	158	58,30
44	TAGGGAAGAGCTCAAGGCAGGTCAACTCGCACTGTGAGGGTCACAT<br>GGGCGTTCGGCACTACCGACACGAACCTCAGTTAGCGTACATCCTAC<br>CAGAGGTCTGTGGCCCCGTGGTCAAAAGTGCGGGTTTCGTATTTGC<br>TGCTCGTCAGTACTTTCAGAATCATGACCTGCACGGCAAAGAGACG<br>CTTGTTGTGGAGCTCGACATGGCAACAACGCGACGGATCTACGTCA<br>CGGCGAGAATAGTGTAAACGAAGCTGCTGACGGCGGAAGCGTCAA<br>A	277	89,9	20	2	164	59,21
45	GGGGTCTGTGAGTTGTCATTCGCGAAAAACATCCGTCCCCGTGGGG<br>GACAGTCACCGACGCCGTTTTGTAGAAGCCTAGGGGAACAGGTTGG<br>TTTGACTAGCTTAAGAAAGTAAATTCTGGGATTATACTGTAGTAATC<br>ACTAATTTACGGTGAGGGTTTTATGGCGGGTCTTCGCAAATTCAAGC<br>CGGGTGATTTCAACAGATTTTGCTGACGGTTTAGGCGCACTATCCCC<br>TGAACTACAAATTAGAAAATAGCGCTCCTCGACGGCTA	271	78,5	20	4	162	59,78
46	GAATTACCTACCGGCCTCCACCATACCTTCGATATTCGCGCCCACTCT<br>CCCATTAGTCCGCACAGGTGGATGTGATGCGATTGCCCGCTAAGAT	271	69,1	20	4	132	48,71

Number	Random sequence	Lenght (176-280pb)	Lowest Energy State	Total Number of Structure	No. Hairpins	No. Paired Nucleotides	% No. Paired Nucleotides
	ATTCTAACGCGTAACGCAGATGAGTATTCTACAGAGTTGCCGTACGC<br>GTTGAACGCTTCACGGATGATAGGAATTTGCGTATAGAGCGCGTCA<br>TCGAGGGGTTATACACCCGTAGACTACAACGGGCCCGGCTCAATCA<br>GAACTCGAGTGCCTTGAATGACATACTCATCACTAAAC						
47	ATTCTCAACAGTCAATCGAGCAAGTCCATTACCAACGAGTGCGTTGC<br>AGTTTCATTCTCTCGCCAGCACTGTAATAGGCACTAAAAGAGTGATG<br>ATAGTCATGAGTGCCGAGCTAAGACGGCGTCGGTGCATAGCGGACT<br>TTCGGTCAGTCGCAATTCCTCACGAGACCCGTCCTGTTGAGCGTATC<br>ACTCCCAGTGTACAAGCAACCCGAGAAGGCTGTGCCTGGACTCAAC<br>CGGATGCAGGATGGACTCCAGACACGGGGCCACCACTC	271	79,6	20	4	158	58,30
48	TTCACGCGTAAAGCAAGAACGTCGAGCAGTCATGAAAGTCTTAGTA<br>CCGCACGTGCCGTCTCACTGCGAATATTGCCTGAAGCTGTACCGTTA<br>TTGGGGGGCAAAGATGGAGTCCTCCTCTTTTCATAATTGTACTGACG<br>ACAGCCGCGTTCCCGGTTTCCTCAGAGGCTAAAGAATAAGGGCTTA<br>TTGTAGGCAGAGGGACGCCCTTTTAGTGGCTGGCGCTAAGTATCTT<br>CGGACCCCCTTGTCTATCCAGATTAATCGAATTCTCTCATT	273	69,8	20	4	160	58,61
49	TAGGACCCTAGTAAGTCATCATTGGTGTTTGAATGCGACCCCGAAG<br>AAACCGCCTAGAAATGTCAATGGTTGGTCCACTAAACTTCATTTAAT<br>CGACTCCTAAATCGGCGCGATAGGCCATTAGAGGTTTAATTTTGTAT<br>GGCAAGGTACTCCCGATCTTAATGGATGGCCGGAAGAGGTACGGA<br>CGCGATATGCGGGGGTGAGAGGGCAAATAGGCGGGTTCGCCTTCG<br>CCACGCTAGGGGGCGATTCTATAAGAATGCACATTGCGTCGA	272	76,2	20	3	166	61,03
50	TACATAAGATGTCTCGACCGCATGCGCAACTTGTGAAGTGTCTACTA<br>TCCCTAAGCCCATTTCCCGCACAGTAACCCCCGATTGTGTCCGCATCT<br>GATGCTACCCGGGTTGAGTTAGCGTCGAGCTCGCGGAACTTATTGC<br>ATGAGTAGAGTTGAGTAAGAGCTGTTAGATGGCTCGCTGAGCTAAT<br>AGTTGCCCACAGAACGTCAAGATTAGAGAACGGTCGTAGCATTATC<br>GGAGGTTCTCTAACTACTATCAGTACCCGCGTCTCGACT	272	70,5	20	2	158	58,09
51	CTGCCGCGGCTACCTATCGCCTGAAAGCCAGTCGGCGTTAAGGGGT<br>GCTCTGTCCAGGACGCCACGCGTAGTGAGACTTACATGTTCGTTGG	278	91,6	20	3	170	61,15

Number	Random sequence	Lenght (176-280pb)	Lowest Energy State	Total Number of Structure	No. Hairpins	No. Paired Nucleotides	% No. Paired Nucleotides
52	GCTCACCCGACTCGGACCTGAGTCGGCCAAGGACGCACTCGAGCTC TGAGCCCCGCTGTCGAGAAGTATGCATCTCGCCCCCGCAGCTTGCCA GCTCTTTCAGTATCATGGGGCCCATGGTTGAATGACTCCTATAACGG ACTTCGACATGGCAAAATCCCCCCCTCGCGACTTCTAGAGGAGAAG AGTACTGACTTGAGCGCTCCCGGCACTTCGGCCAAGGAAGTCACCA ATTTCTTGTTCCCGAATGACACGCGTCTCCCTGCGGGTAAATCGCCG ACCGCAGAACTTACGAGCCAGGGGGAACAGTAAGGCCTAATTAGG TAAAGGGAGTAAGTGCTCGGACGGTTCAGTTGTAACCATATACTTA CGCTGGGCCTTCTCCGGCGGATTT	208	67,8	13	4	118	56,73
53	TTACCGTCACCAACCACGAGATTTGAGGTAAACCAGATGAGCACAT AGCGGCGCTATCCGGCTATCTCCAAATTGTAACATACCGTTCCATGA AGGCCAGAGTTACTTACCGGCCCTCTCCATGCGCGCGCCATACCCCC CCAGTCCCCCGGTTATCCCTCCGAGGGGAGGGTGAGCGATCCTCCG TTAACATATTGTTACCAGTG	206	53,9	4	3	116	56,31
54	ACGTAGCTATGTATTTTGCACAGGTGGCCAACGGGTTCCACACTTCA CAGATGGTGGGGATCCCGGCAAAGGGCGTGTATTTGCGGTCCAACA CAGGCGTAGACTACGATGGCGCCTACTCAGACGCAGCTCGTGCGGC GTGAATAACGTACTCATCCCAACTGATTCTCGGCAATCTACGGAGCG ACACGATTATCAACGGCTGTCT	208	58,1	17	4	126	60,58
55	AGCAGTTCTAATCTCTTGCCACGGTCGTAAAAGCCTCCAAGAGATTG ATCATACCCATCGGCACAGAGGTGACACGGCGCCGATGGGTAGCG GACTTTGGGTCAACCACAGTTCGGCAGGGGACAGGCCCTGCGGCGC GCATCACTCTGTATGTGCAACGTGCCCAAGTGGCGCCAGGCAGGAC TCAGCTGGTTCCTGCGTCAGCTCGA	209	84,7	15	6	124	59,33
56	GGCTGGGCATGACAGCTCTTTGAACATGGGCCGGGGGCCTCGAAC GGTCGAGAAGCCCATAGTACCTCGGGTACCAAGTTGCGCAGGCTAT AGCTTGAAGCTGTACCGTTTCAGGGGGGGAGCCCTGATGGTCTCTT CTTCTGATGACTCAACTCGCCAGGGTCGTGAAGTCGGTTCCTTCGAT GGTTAAAGATCAAAGGCTCAGAGT	208	76,3	15	7	130	62,50
57	GCGGACTGGAGCGCCCATCTAGCGGCTCGCGTCTCGAATGCTCGGT	208	61,2	12	4	118	56,73

Number	Random sequence	Lenght (176-280pb)	Lowest Energy State	Total Number of Structure	No. Hairpins	No. Paired Nucleotides	% No. Paired Nucleotides
	CGCCTTTCACATTCCGCGAAAATCCATACCGCTCATTCACCAGGTTGC<br>GAAGTCTACACTGGTATATGAATCCGAGCTAGAGCAGGGCCCTTAA<br>AATTCGGAGTCGTTGATGCTCAATACTCCAATCGGTTTTCTCGTGCA<br>CCACCGCGAGTGGCTGACAAG						
58	GGTTTGACATTGAGTAGCAAGGCAGTTCCGGGCTGAATGAAGCGCC<br>GGGAAAGGTACGCGCCCGGTATGGCAGGATCAAGGGGCCAATACA<br>GAGGCTGCACCCTCACTCGGATGGAGGCAAACGCAGAACAATGGTT<br>ACTCCTTCGATACGTGAAACGTGTCCCACGGTAGCCCAAAGACTTGA<br>GAGTCTATCACCCCTAGGGCCCTTTCC	211	62,2	15	2	104	49,29
59	CGGATATAAACGCCAGGTTGAATCCGCATTTGGAGGTACGGTGGAT<br>CAGTCTGGGTGGGGCGCGCCCCATTTATACCGTGAGTAGGGTCGAC<br>CAAGAGCCGCAAGATGCGACGGTGTACAAGTAGTTGTCGACAGACC<br>GTCGTGTTTTCATAACGGTACCAGGATCTTCGGGCCGTGTCAATCAA<br>GCTCGGATTGCGGTGTCTACTCCGT	210	70,2	15	3	154	73,33
60	CCTGCGGCTACCCACGGCCTGTAATCCACCTCGAGTCAAGCCATCGC<br>CTCTCCGGGACGCCGCATGAACTGATACGTACACCTTGCGCGGGTT<br>CACCGCGGTCCGTTCAGAGTCGTCCAAGGGCACAATCGAGCTCCCA<br>TCCGTACGCTCGGCTAACTTGTACCCGACCCCCGGAGCTTGGCAGGT<br>CGTGGGGTGTCATGGAGCCTCTGGTTC	213	74,2	14	3	130	61,03
61	ATCCCGTGGGATATCGAGCTTCGTCTTGATAAAGCCCCCCGCTCGGG<br>TGTAGCAGAGAAGACGCCTACTGAGTTGTGCGATCCCTGCACCTCA<br>GCCAAGGTAGCTACCAATATTTAGTTTCCGAGCCTCGCGACAGACCT<br>CCCGCCTAGATTGCCACGCGTAGAGCTAGCGAGCCAGCGGAAAGC<br>GTGACGCGCTTTCAAGCG	203	60,3	19	4	120	59,11
62	TGGCGAGTATGTGAACCAAGGCTTCGGACGGGACTATATACTTAGG<br>CTCGATCTCGCCCCGAGAACTGTAAGCCTCAGCATCTACGGGTTATG<br>AGGTTAGCCGAAAATGCACGTGGTGGCGCCCGCCGACTGCTCCCTG<br>AGTGTGGCTCTTCGTTCCGTCAACGCCCGACCTTCATCGCGGCCGAT<br>TCCTTCCGCGGACCATGCCGTCC	209	70,4	8	3	140	66,99
63	TGATACTTCGGCCATGTTTCCGTTGTAGGAGTGAGGCCACCTGGCTT	211	72,9	18	3	128	60,66

Number	Random sequence	Lenght (176-280pb)	Lowest Energy State	Total Number of Structure	No. Hairpins	No. Paired Nucleotides	% No. Paired Nucleotides
	TGCGCCGTGGTTCCAATGAAAAACCTATGGACTTTGTTCAGGGTGG<br>CATCGGGAATCTGAACCCTCAGAAAGTGGGGATCCCGGGTATGGGC<br>CTTTATCTGCGGTCCAAGTTAGGCGTAAGGCTGCATGCTACCTTGTC<br>ACACCCACGCCGCCCGGGGTAAATA						
64	TGGGAGGCGTGCGACCTGGCTCCAGGCGTTCCGCGCCGCCACGTGT<br>TCGTTAACTGTTGACTGGTGGCACATAAGTAATACCATGGTCCCTCA<br>AGTTCGGCTCAGTTACCTCGAGCGTTATGCCTCAAATGGCGCAGGA<br>CGGCATTGGCTGCCCGACACTAGCTGGTGTTCGGTTCGGTAACGGA<br>GAGTCCGTGCGGCGATGTCATTAA	209	72,5	20	2	134	64,11
65	TGCATTTGAAACGCGCCGTACCGACGCTGGGCAAGTCAGTGCAGGC<br>TCCCGTGTTAGGACGAGGGTAAACATACAAGCCGATAGAAGATGG<br>GTAGGGGCCTTCAATTCGTCCAGCACCCCACGGCTCCTCCGAGAGCT<br>AGTAGGGCACCCTGTAGTTGGAAGGGGAACTATTTCGTGGGGCGA<br>GCCCACACCGTCTCTCCTGCGGAAG	208	67,9	20	3	130	62,50
66	ACTTAACACGGTAGGGAGCTGGAGTGGTTTCGAACGATGGTTATTA<br>ATCCTAGTAACGGAACGCTGTCTGGAGGGTGAGTCTGACGGAGCGT<br>AACTCGATCGGTCACTCGCTATTCGAACTGGGCGAAAGATCCCAGC<br>GCTCATGCACTTGGTCCCGAGGCCTGACCCGATATATGAGCCCAGA<br>CTAGAGCGGGGCTGTTGACGTTTGG	209	62,5	20	2	120	57,42
67	AGTTGAAAAAATCTATTATACCAATCGGCTTCAACGTGCTCCACGGC<br>GGGCGCCTGACGAGGGGCCCACACCGAGGAAGTAGACTGTTGCGC<br>GCTGGGGGTAGCGGCGGCTAACCAAGACGCCTGCCACAGCAGCAG<br>TATCAAACCCGTACAAAGGGAACATCCACACTTCGGTGAACCGAAG<br>CGCGGCATCAGGGTTT	199	66,9	9	3	114	57,29
68	CCTTTTGGATACCTGATACAAAGCCCATCGTGGTCCTTAGACTTCGC<br>ACACTTACACCCGCACCGCGCGCATGTGGAATTAGAGGCGAAGTAC<br>GATCCCTAGACCGACGTACGATGCAACTGTGTGGATGTGACGAGCT<br>TCTTTTATACGCTTCGCCCGCCGGACCGGCCTCGGCATGGCGCGGC<br>GGTGCACAAGCAAATGACAACTA	208	58,8	11	3	128	61,54
69	ACCACCGTGTATTCGTTATGGCACCAGGCAGTTTAAGCCGGGACAA	207	64,6	6	3	112	54,11

Number	Random sequence	Lenght (176-280pb)	Lowest Energy State	Total Number of Structure	No. Hairpins	No. Paired Nucleotides	% No. Paired Nucleotides
	TGGGGGCCGCAATACACAGTTTACCGCATCTTGGCCTAACTGACATA<br>CTGCCATGGACGACTAGCCATGCCACTGGCTCTTAGACAGCCCGATA<br>CAGTGATTATGAAAGGTTTGCGGGGCATGGCTACGACTCGCTCAGC<br>CACGTGCGAGGGGAGAAACCT						
70	TTGCGCATTTGTATGTTCACCCGTCTACTACCCATGCCCGGAGGTTAT<br>GTAGGTTGTGAGATGCGGGGGAGGCTCTCGACCTTCCCGTGGGAC<br>GTCAACCTACCCCTTGATAGAGCACCCCGCTCGGGCATGGCAGTGA<br>GTACGCCTTCTCAATTGTGCTAACCTTCGTCCTTATCAAAGCTTGGAG<br>CCAATGATCAGGATTATTGCCT	209	65,5	9	2	126	60,29
71	TGCGACAGACCTCCTACTCACAGTCGCTCACATTGAGCTACTCGGTG<br>GGCCATCAGCTTGACCCGGTCTGTTGGGCCGCGACTACGTGAGCTA<br>GGGCTCCGGACTGCGCTGTATAGTCGAGTCTGATCCGGCCCCCACA<br>ACTGCAAACCCCAACTTATTTAGATAACATGGTTAGCCGAAGTTGCA<br>CGGGGTGCCCACCGCGGAGTCCT	209	75,7	11	3	136	65,07
72	CCCCGGGTGTCCCTCCTTCATCTGACGACGAGCAGCCGCTACCGCCA<br>TCGATTGATACAGGGGACGGTGATGTCGTCATAGATTCGGCACGTT<br>ACCCTTGTAGGTGTGGAACCACTTAGCTACGCGCCGAAGTCTTATG<br>GCAAAACCGATGGACAATGATTCGGGTAGCACCAGGAGTCCGTAGC<br>ACGTGCATCCCGACGTGGCGCGCGT	210	67,6	16	4	118	56,19
73	ACAGCTTAACCACCGCTTCATGCTAGGGTGCTGGCTGCATGCTAGGT<br>TGACACGCCTGCACTGCTCGAAGAAAATATACGAAGCGGGCGGCCT<br>GGCCGGAGCGCTACCCCATCGACGCGTGCTCGAATACTGTTAATTG<br>CTCGCACATGAGCAAAATAGTAGAGCGTCACTTTCAGCCCTCTTATC<br>CTCGGCGATGTGTGTGAAATGGCG	210	68,8	14	4	122	58,10
74	TTGATCTGGATTGACTCTATGACGGTACCTGCTGATGGGTAGGGAG<br>ACCCGGAATCTATCGGCCTATGTCACTGAAACTATCCAAACGCCCCG<br>TGTCGATGCTGAACGTATCGACGCACGCCCCCCTCCTTGAAAACGCA<br>CAATCATACAACTGGGCACATGATGCGTACGCCCGTCTAGTACACCC<br>ATCTCTGTAGGCCCAGTTCAA	208	63,5	3	3	124	59,62
75	GAGCTGGAAGGGCACCCTCCACTTGGTCAGGCGATATCCTGGTAAG	202	63,9	14	3	114	56,44

Number	Random sequence	Lenght (176-280pb)	Lowest Energy State	Total Number of Structure	No. Hairpins	No. Paired Nucleotides	% No. Paired Nucleotides
	GCAAGCCCGTACCGTGATTCATGCGGCAGGGGTAAGACCATTGGAA GTAGGGATAGTCCCGAACCTCGCTTACCACTGCCAATAAGGGGTCC CTGTCTGAAGGATGAGTGTCAGCCAGTGTAACCCGATGAGGAACCC AGAAGCCGAACTGGGCCA						
76	GACAACCCGGCGCTAACGCACTCAAAGCCGGGGCGCGACGCGACA TAACGGCTAAGAGTAGCCCCGGGGTGTAGACCTTTGGGGTTGGATA AATCTGTCGTGGTAACCGGCTTCAACGACCCGCACGCGTGGCACCTC AGGAGGCGCCCGCAGGGGGGAAGTTTTCTGCTATTCGAGGCCGTTC GTGGTAGCTAGTTGCGTTCCTAGC	208	82,7	14	4	128	61,54
77	CGCTACAATTGTTTCTATGCCGAGTAATGGGAACAACCACACCATAG CGATTCGACGCGGCGCCTCGGAATACCGTTTTGGCAGGCGCTTGCT AAGGCCATCGCGAATTCCAGGTATCGTGCACGTAGCGTAGGGCCGC ACGCAAGTCGAACTGCTGGGGAACCGCGTTTCCACGACCGGCGCAC GATTCAACTTCGCCGACGTGACGACGTTCCT	216	73,8	19	3	130	60,19
78	GCTAATGCCTCGCCCGCCGGACCCCCCTCGTGATGGGGTAGCTGGG CATGTCCTTGTGAGATATAACGAGAGCCTGCCTGTTTAATGATCTCA CGGCGAAAGTCGGGGGGACAGCAGCGGCTGCAGACATTATACCGC AACAACACCAAGGTGAGATAGCCCCGTAGTCGACTACGCATCCCTCT AGGCCTTACTTGGCCGGATACAGT	209	68,7	13	3	116	55,50
79	GACTTTGACACGTTTGTGGGCTACAGCAATCACATCCAAGGCTGCCT GTGGGGGAAGCAACTCTTGGGTGTTAGTATGTTGACCCCTGTATTA GGGATGCGGGTAGTAGATGAGCGCAGGGACACCGAGGTCAAGTAC ACCACCCTCTCGTGGGAGGTGTTCCAGATCACCATACCACCATACCG TTCGAGCATGGCACTA	201	60,5	16	3	122	60,70
80	TCTGCGCCGTCCCCATCCTGGTAGTCATCATCCCTATCACGCCTTCGA GTGACTGGTGACGGATATCCCCCACGAATGGAGATCCTTCTCACTGA CGGTCACATTGGGGTGCTCCTAGGCTCTTCCGCTTGGCCGGGTCTGC TGGGCCCCCGTGCCCGGAGTTTCGGCGCTGCGCTGCCGAGAGCCGG CCATTGTCATCGGGGCCTCACTT	211	77,0	20	4	134	63,51
81	GAGGATACCCCGACCTATTTTGTCGGGACCACTCGGGGTAGTCGCT	203	62,1	15	4	116	57,14

Number	Random sequence	Lenght (176-280pb)	Lowest Energy State	Total Number of Structure	No. Hairpins	No. Paired Nucleotides	% No. Paired Nucleotides
	GGGCTTGTGCACCGTGAAGTCCTCCGCCGGCCTCCCCGCTACAGAA GACGATAAGCTCCGGCAAGCAGCTATGAACAACGCAAGGATCGGC GATATAAACAGAGAAACGGCTGATTACACTTGTTCGTGTGGTATCG CTAAACAGCCCCGCGGAGCC						
82	TTATGCCATACCCGTCCGCGGAGCACTCCGGTAGCGCTTATGGTCCA TAGGACGTTCATCGCTTCCGGGCGTGCGCTCTATTTGACGACCCCTT GGCGCACAGGTGCTGGCCACGAGCTAAATTAGAGCGGCTGCACAG CTGTAAGGTCCGTCACGCAGACGGCGGCCCGGGGAGACCACTGGC CCATCGACCTGTACGGGAACCCTC	208	73,3	20	3	132	63,46
83	TGTATCGTTCTCGGACGGAGAGATAACTACAGTGCCGCTTACAGCCC CCCTGTCGTCGCCGACGTCTGTAATGTGGCCTCGTTGTGATTCCACC CTATTGAGGCATCGACTGATGCGGGAGGAGATCTGGAATGAACTG GCCTATGCGACAGAAACTGTGCAGCCACCCAATCTCCTTAGTGTAGG TTCTGACCGATTCGTGCTTCGTT	209	64,4	7	5	132	63,16
84	GAGAACTCACAATTTAACAACAGGGGACATAAGCCCTACGCCCATG ATCTACTGGCGTCCCTGAGGCTGCAGTTCATGTAATGGGACAGTATC CGCGGCAAGTCCTAGTGCAATGGCGGTATTCTACCCTCGTACTGTG GTAGAGGCGACGCGGGTGCGGCCATCACTAATAGGGATACTGGGA AGGCTCACAGGCCTCCGCCTTTAG	208	64,8	20	4	114	54,81
85	GCGGTGCTTACTCTTACATAAAGGGGCTGTCAGTATTACCCCGCGA GGATTCGAAAAGGTGAGCCGACCCGGCCGATCCGGAGGGACGGGC CTCAAAGCCGCGTGACGACGGCTGTGGGCCCGTAACAGAATCCCCG CAGTGAGCTCCCGTGGGCGTCGGTTGAACAGCCCTGGTCGGCCCCA TCAGCAGCCCGAATACGTCGCTTTAC	209	78,8	13	2	134	64,11
86	GGGTCCCGGGCCGGGGCGCGATGCCTTGCAGAAATCGAGGCCGTT CGTTAATTCCTGTTGCGTTCGTACCGCCTATATTTGTCTCTTTGCCGG CTTATGTGGACAAGCACAGCATAGCCATTTATCGGAGCGCCTCCGTA CACGGTATGACCGGGCGCCTCGTGAGACCAATGCGTATACCAGGTG TCCTGTGAGCAGCGAAGGCCCA	208	70,1	19	4	136	65,38
87	TACGCGAGATACACTGCCAGGAATCCGCGTGACTACGAGCCGTGGC	212	93,5	15	2	132	62,26

Number	Random sequence	Lenght (176-280pb)	Lowest Energy State	Total Number of Structure	No. Hairpins	No. Paired Nucleotides	% No. Paired Nucleotides
	GGATTTGGTCTGGCTGTGGTCTAGACATTCCAGGCGGTGCGTCTGC<br>TCCCGGGTGCCTCTGGTGGCTGGCTAGATGGACTAGCCGCTGGTAG<br>ACACACCATGACCCCGGCTCTCCATTGATGCCACGGCGATTGTCGGG<br>GAGCCAGCAGCGGCTGCAGACATCAGA						
88	TCGGAGTAACACTAGCGTGCGATAAGTCCCTAACTGACTACGGCCTT<br>CTGTAGAGTCAACTTCACCACATACGCTGTCTCTGGCACGTGGATGG<br>TTTAGAGGAATCAGATCCAAGTCTGGCTAACCACCAAGCAGGTCTT<br>GAGTCTAAAATTGTCGTCTCCTGCGTACGGGATGGGGGTACTAGGT<br>GACTGCAGGGACTCCGACGTTA	208	62,2	18	5	130	62,50
89	TGTACGTTGCCCCGTCAGAGGCGCCGTTCAGGATCACGTTACCGCG<br>AAAAGAAGGGACCAGGAGCTCTTCTCCCCTGCGGCCACGCCTGTAG<br>AGATTACACCATTAACCCTCCTGAGAGCCGGGAGGCGGGAATCCGC<br>CACGTATGAGAAGGTATTTGCCCGACAATCAATACCCCGGGCTCCTA<br>ACCTTTTCCACTCGCTTGGG	205	66,0	11	4	110	53,66
90	CCGGCTAGGCCTCTCTGCCCGGAGTTTCGGCGGACTGGTGCCGACG<br>CGCGGGCATAGTTTTAGGGGGGTTATTCGGGGGCAGTGGCAGCCA<br>ACATCTCGGGTCCTGCCCGGCCGGTCTACGCGCTAATACAGCGAATC<br>GCCGAGGACCCGGCGCCACGCAATGGAACGTCCTTAGCTCCGGCAG<br>GCAATTAAGGGGAACGTATGCATG	208	84,2	16	3	138	66,35
91	GCGCAAAAAAACAGAGAAACGGGCGAATGGACCTCTTCCCCGCGTA<br>TCGGAGAATGGCCTCGCGGAGGCATGCGCCATGCTGGCGCGCGGG<br>GCACTCTCGCTACCCATATGGTCCACAGGACACTCGTCGCTTCCGGG<br>CTTGCCCTCTATGCGCCGGTCCTCAGCCGCGCTCATGCCCGGCACCG<br>CCACAACCAGACCGACACCAGATGTGTAAGGTCCGCCACGCAGACG<br>AGGCCGGCCGGAGACCACCGAGCGTTCTACCAGGTCGGCGA	272	101,0	20	3	162	59,56
92	CCACCAGTGAGCTACTGGGGCCGAGGGGTAACCACGGTGCCGCTA<br>AGAGCCCCTCGGTCGACGCGGGCGACTGCACTCCTGCCACATCGTG<br>ATCGCTCGCTATTCAGGGGTTGACCGACGCCGGAAGACTTCTCGCTT<br>GAAGTGCTGTATGCGACAGGGTGCGTGCACCTACCAAACCTGCTTA<br>GACTAGGTTCAGACCGGTTGGAGGTGCGTCCAGATCTCAGTTTTCG	232	81,0	20	6	132	56,90

Number	Random sequence	Lenght (176-280pb)	Lowest Energy State	Total Number of Structure	No. Hairpins	No. Paired Nucleotides	% No. Paired Nucleotides
	TC						
93	ACCAGGGGGCCCACGCCCTACCCCCATGATCCACTGACCTCCCAGAC<br>GCTGCAAGACTTGCAACCGGGCAGACTCGGCGGCAGGTCCTAGTGC<br>AGCGGGGCTTTTCTTCCGTGGTCCTCGAGAGGAGGGGTCGCCGGTC<br>CGGACACCTCTGATGTCCTGATTGGGAGGACCGTCGGCCCCCCGCC<br>CTTAGGCGGTGCACTCAGTTCCATAAACGGGCTGTTAGATATGGGG<br>CCCG	235	103,9	20	6	148	62,98
94	TGGATTGGAAAGGGTGACGGAACCCGCCCGAACGGGAGAGACGG<br>GCGACTAGGCGCCCTGAGCACGGCTGCGCGCCCGTATCAAGCTCCC<br>CCCTATAGGCCCCGGTCTCCGTCGGTCGTAGAGCGCAGGACGGGCC<br>GGGGGGATGCGCGACAATGTCGCTTAGCCGCCCCTGGGCCGCGGT<br>CCGCTACCTTGCAGGAATCGAGGCCGTCCCTTAATTCCCCTTGCGTA<br>CGTGCCGCGTTTCTTCGACCCTTTGGCCGCTCCCCTGG	266	113,3	20	6	178	66,92
95	GGGAGGGACGGATAGCCTCTTACCCGTGCCCCACCGTTGGCGGCAC<br>GACCGCACGCCCCGCGTGAGCCATTGGTAAACCCCGTGGCCTGTGA<br>GCGGCGAAGGCCCTAACGGGAGATTCGCGCCCACAACTCGGCCCG<br>AATACGGGTCCTGGCAACGTTCGTCTGGGTCTGGTCTACACAATGC<br>GGGCGGTGCGTCTGCTCTGGCCAGCCTCCAGTGGCTCGCATGATAG<br>TGCACCCGCTGGTGATCACTCGATGACCCGGGCTCCCCGT	269	119,4	20	5	180	66,91
96	TGCAGCCACGGGGATTCTTCGGGACCGACCTGCGTCCGGCATCGTG<br>GGCACAGTGAAGTACTAGCAAGCGTTAAGTCCCGAACTGGGTGTGG<br>CCTAGCGGTGAGAGAACTTCACGACACGTCCTGCCGCACGCGCGAG<br>GTACGTTTGGACGGTACTGAATGGACTCTGGTCAACCTTCACACCG<br>GTCTAGAGTCGAATGCGCAGGTCAGCCGGGTGCGAGCCGGAAGTT<br>CTAG	233	80,4	20	4	152	65,24
97	GTCACTGGAGGCTCTGCGACGTTCTGAGCGTTGGACGGAGTGACCC<br>GCGACCCAGGATGAGGTCGCCCCGAAAAATAGGCTCCTGCAGCTCT<br>CCTCGCGAGCGGTCTGGTGTATCGAAAGTACAGGACTAGCCCTCCT<br>GGCAACCGCGGGCCGGGAGTCCGAGACGTCACTCAAGGTATATGC<br>CCGGCAACGTATGCCCC	200	69,8	20	5	120	60,00

Number	Random sequence	Lenght (176-280pb)	Lowest Energy State	Total Number of Structure	No. Hairpins	No. Paired Nucleotides	% No. Paired Nucleotides
98	AGCCACCTAACTATTCCCTGTGCCTTGTGGGGGCCTGCGCTGTCTGC CCGTCGAACCGTGGGACTCGCGCCAGCGCGCAGGCTTGGATCGAG GTGGAATCTCCGGGGCCTGAGGCCACGAGCGTCTGGCGTCCTGGCC AACCCCCCTACGCGCTGTTATAGGCAATCGGCGGGAACCCGGCGCC AGGGGGTGGAGCGACCTTAAGTCGGG	210	89,9	20	3	132	62,86
99	GACGGTATTAATCGGAAGGAGTGTTCAACGCAATGAAGCCGCAGG GTTGGCGTGGGAATGGTGCCTCTGTCCAAGCAGGTAAGGGCACGG GGCCGCAACCGTCCCCCAAGCGTGCGGGGTGCACTTCGCAACGATT TCGGGGTCCGGAGACTCGCTGCTTTCGGAATTCGCGCTCAAGGGCG GGTATTGAGCCAGGCTTACGCCCAGGA	209	74,3	20	4	130	62,20
100	ACGTAGCAAGGTGACCCAAACAAGGTGCATCTTGCCCGCGTTCCAC ACGAGTCGAGTCGGAGGTTACGGAGCATAGTAACACGTGGGCGGC CAGTGGTCGGCTGCTGCACCCCTGCCGCAACGTCGAAGGCCCCGGG TTGGACTGGCTGGACCCATGCCGTGACACCCGTCACACTCCATTACC GTCCGCGGGTCACGGC	200	75,2	18	3	130	65,00
101	TTGTCGTGGACCGGATTGCCATTCTCTCGGTGTATTGCGCAGGCCGG CGCGCGGGCCCCATGCAAACCTGTCATAGCTTACCTGACTCTACTTG GAAGTGTGGCTAGGCCTCTGCCCACGCGCCTGGTCGGCCCTCGCTT GCTTTTTAGGACCGGATGAACTGCGGGGCGCTGCAAGAATCCCTAC CTGCCTCACAAGGC	200	68,5	10	4	112	56,00
102	GCTGGGTCCTACTCCAGCGGGGCGTTTTATCTAAACACGATGAGAG GAGCACTCGTCAGGCCACATGGCTTTCTTGTCCTGGTCGGATCCACC GTTGGCGCCCGACCCCCCCGCTCCGTAGTGAGTTCCTCGTCCGAGCC ATTGCATGCCAGGTCGGCAGACAGATAGCGGACCCGGTATACCCCC GGAGGCCATAGACGCACAGGTT	208	72,6	18	4	118	56,73
103	GGGATCCTGAGCGGGGTCGCGCGTCCGAACCCAGCTCCACTTTAGT GGCCGCGGGTTCTGGTCCCCCCGGGCCGCGGAACCGGTTGGGGCC ATGTGCGACAATACCCGTTAGTCGCCCTTCGGGCACGGTCCCCCTGC TCGGTGGTATACGGTCCCTGCCATAATTAGCCACCCTCATAAGTTGC GCTACTTCCGCGACCCAAGCGCACCCTTACCACGGAGACAG	226	91,2	16	3	146	64,60

Number	Random sequence	Lenght (176-280pb)	Lowest Energy State	Total Number of Structure	No. Hairpins	No. Paired Nucleotides	% No. Paired Nucleotides
104	GATCGTCCGATCCCGTGCCGCGGCCTTGGCAGGGGGTTCGCAGGCC CCACCCCAAGCGATGCTGAAGGCTCAGGCCACACAGGCACAAGTGC TATATACGCGAGTTCCCGCCCTTAACCTGGACCGAATGCGGGACCAT GCATCGTACCACTGTGTTCGTGCCACCTAGGACGGGCGCAAGGGGT GCATAGCTCAGCCAGGAATGCCTCGTATCATTGTGCACCC	225	83,4	20	6	138	61,33
105	GCCGGTCACCAGCCGACGACGTGCGGACGGCGTTGCGACTTGCCG GGCCCGACCTCACCGCCCTGGGTACCGCACACTGGGCAGTGCGAGG TAAAGCCAGTCGCCCAGTGCCGATCAGCAGCTGACGTAACGGTAAG AGGCCCACAAAATCGCACCGCCGGCGCCCCCTGGGTACTTTACGTC AGCATCGGGTGGACTGGCATGAACCTTCACTCCCAGGCGGAAACG	228	94,1	12	2	146	64,04
106	GGTGCGCGGACGAGCGAGCAGCAAACGAAAATTCCTGGCCTGCTT GGCGTCTCGCGTCCCTCTTGGGGATCGAGGAAATGTTTCGCGACCG AGGGAGAGGTCGCCCTGCGAAATAGATTTGCGCTACTGTCCGCGTG AGGAGTCCGGTGTAGCGAAGGATGAGGGCGACCCTAGGTAGCAAC CGCCGGCTCCGGCGGTGAGGCATCACTCAGGAAGCAGGCACGGAA AG	229	90,5	20	4	144	62,88
107	GCACGGTCTAGCGGACCGTCTATCGGCTGGGCCAAATGGGGCGCTC CGATATCAGCGTGTCCAGCCTTAGGACTCGGCTCAGCGCGCTGGCC TGGGTCGAGATGAAATCACCGGCGCCCAAGACCAGGCGGGCCCGC CGCGTTGGCTAACCCCGGTACATCTTGTAATCGACGTTCAGAGGAA AGCCTGTGCCAGAGGGACGAGTCACCACGTACCAGCGGCGAC	225	86,1	20	5	132	58,67
108	AACGATCGGTCGGACTATTCATCGCGGTGGTGGCGCTCGGATCGCG CGGGAAAGGTGCTTGTGTCCCGGCAGGCTAGGGTATGACGCCGGG GCGCTGCCCCAACCGTTCAGCGTGGGGTTTGCTACGACTTCCGAGT GCTGCGTGTGCGAGACCACGTTATGTGCGCACAAGGCCGGCAATAG GACGTGGCCTTCGGGCTAGTACGTAGCGTGGTCGCACAAGCA	225	103,7	17	3	144	64,00
109	CAGTAGATCCTCCCCGCGCATCCCACCTATCAAGTTGGTCCTATAGC AGCACGGTCACACGCGGACGGGCAGTGGCCGGTAGCCACACGCCT GCCGCGGCGCTCAACGACCGGGACTAGAGAGGCGGGGACCATGGC GTGTGACCCGTCATGCTCGAGTTCGGTCAGAGCGTCACCGCGAGTA	222	83,5	20	2	134	60,36

Number	Random sequence	Lenght (176-280pb)	Lowest Energy State	Total Number of Structure	No. Hairpins	No. Paired Nucleotides	% No. Paired Nucleotides
	GTCGACTGCTTTCCCAATCTCCGAGCGATTTAGCGTGGC						
110	GGCCCCAGGGGACCCACAAAATGCGGTCGCAGCCCACCCGACCGTA<br>CACGGAAAGGAGGGTCCCCGTACGCCGACGCACCTGTTCGCACGTC<br>GTATGCATAAACGGGCCGCACGAACCAGAGAGCACAGGGAGGACC<br>TCTAGCTCCTTTACAAAGCGCAGGTTCGCCCGCCGCCGGGATGCCTT<br>ACCTAGACGCGATGACGGGCGTATTCCTCTGGCCTCAACGGTTCC	229	80,4	16	3	130	56,77
111	TGCTTCCGCTGGGATCCAAGGTTGGCGGCCGAGGCCGCCTTTCCGA<br>AGTGAGTCCTTCGTCCGTGGCTAACTGTGCCAGATCGTCTCGCAGAC<br>TCCCGATCCAGTTTAACCCACCAAGCTATAGCCGCACAGGCCCGAAT<br>CCTAAGTCATGTCGCGCGACCGGCCCCTGCTCAATTTCTGTGCTCAG<br>GGGTTCTGGTCCGCCCGAGCGGCGCAGCCGATTAGG	223	77,8	19	5	128	57,40
112	GCCATCTAGTGCACTTGTTGCAAGACTCCTTTCGGACACCTTCTCCCT<br>GCCCAGTGGCGGATGGTGGCGGCTGTTGCCAGCCGGCGTGGAAGG<br>TAGCGGCACCGGCGCGAGCCTAGCGCGCCGTCTCCACCAACACAGG<br>GCTGTCCGGTCGCATAGTGGGGCCCCGCGACGGGGTT	176	76,5	19	3	118	67,05
113	GGCAAGTGGCAGCCCAAACGATGCCGGGGGCTCGCGATGCACACG<br>CTCTGGTCCAATACACGCGTGACCCGGCAGTTATCCTGCACCGGAAC<br>GCCGATCGTGCATCGGGCCAGCGTGATCGTGTCATCTGGGAGGCG<br>GCCGTAGGATAGATAGTTCAATGAAGATGTCGTCCTGCCAGTGTAC<br>GCCTGGGCGTCACCCGCCA	202	82,4	16	3	142	70,30
114	TCCCTGTGCGGGCGGGCCGGCGAGACGCTGCCCCTGATCTCTCCGC<br>CGCTGATAGCGCACACGGGGCAATACCAGCACAAGCCAGTCTCGCG<br>GGGGCGCTCGTCAGCACACGAAAGGGCTTGAGGCACGCCAATTCG<br>CACCGCCGGGGTCGCCTGGGTGTTTTGCGCCACCGTCGGGTGCGCT<br>GGCATGCGCCCTTCCTCCCAG	204	85,6	20	2	136	66,67
115	GGGCATGCGGCTGCGTGGTCAAGGGTGCGGCATTCGTATTTGCCCC<br>CCGTGCTTGCTCTCACGAGCCCGACCTGGAGATCGAGGAGATGCTT<br>CTCGTGGAACCGGACGGCGCGCCAACGCAACGGATCTGCGCTACAG<br>CGCGCATAGCGAGAGCGGAGTTGCCGACGACGAAGGCGACGCTGG<br>GATC	187	71,7	15	3	112	59,89

Number	Random sequence	Lenght (176-280pb)	Lowest Energy State	Total Number of Structure	No. Hairpins	No. Paired Nucleotides	% No. Paired Nucleotides
116	CGTCCGCCGCCACCCGCGGAAAGCATCCGCTCACGAGGCGGGCACCGATTGACACGGTCTTGCAGAGGGTCAGGGGGGTAGGTCAAATTGGGTGGCTTGAAAACGCCGTGTCCGGGGTTAGAGTGCAGTAGACCGCGGTCGGCGGGGACGGCTTTAAGACAGGAGTTCGCAAGACCAGGCGGGGTCGCCGCGACGGCTATTCC	203	76,4	20	2	120	59,11
117	CGGTGGTTCAGGCGTACAATGTCCTGAAGAATATCTGAGAAAGAGGCACCCCTCGCCGCCTAGGATCGCCCACCGCGGTCGGCCGCACCTTCGATCGTCGCGCCCACCCTCCCATTAGCCGGCAGAGGTGGTTGTGTCGCGACAGCCCAGCGTGATATCCTGAGGCGCTGCGCCGACGGAT	181	65,4	14	3	112	61,88
118	GTCCCACGGGATTGCCACGGGCGCCGAGCGCTACGCGGGCGACACGAACTTATGTACGGAGCGGGCCGTCGAAAGGTCGTGCCCTTGCAGTTAGCACGTAGCCCGGCCCCACTAGCACAGCAGTGCCTCGGGCGGCATCCTCATTATTAAGTTTTCTCTACAGCCAAACGACCAGGTGCGCTTCCGCGGAGCGCGGTGGAGA	202	73,2	17	2	128	63,37
119	CTCGCCCACCCGGCAGCTCTGTGACGGGGACTAGAGGGGCGACGACAATCGCGAGTGCCGCGTTATGGTGGTGTCGGGACAGAGCGGCCCTGCGGCCAGTCGTATCCCTTCCCGAGCTCCGTCCGGTTAAGCGTGACACCCCCAGCGTACCCGCAAACCGCGATGGCTGTGCTCGGGGTCGATCGCACGTAGGACGGTCCCC	202	81,0	20	3	128	63,37
120	AGACGCCGGGGCACCAGTTCCCACGCCCAAAGCATAAACGACGGGCAGCCACGAGAGTCCTAGAGCTGGACGTGCCGCTCCTCTGCGAACAACACCTCGGGCTGCGCCGCCGTTGCGCTGCCTAGACGCAGTGCCGCTCCTATCGCACTCGCCTCGACGGCTGCCGCCTCCGCTGCCTCCCTAGGCACCCGGCGGTAGGCG	201	84,5	12	3	114	56,72

**Mean**	234,28	80,25	17,21	3,63	141,82	60,58
**Median**	234	80,0	17	4	142	61,00
**Max**	280	126,7	20	7	182	73,33
**Min**	176	53,9	3	2	104	48,71
