# Supplementary material for: Secondary structure of nrDNA Internal Transcribed Spacers as a useful tool to align highly divergent species in phylogenetic studies
Source: Genet Mol Biol. 2017 Feb 13;40(1 Suppl 1):191–9. doi: 10.1590/1678-4685-GMB-2016-0042 (PMC5452138; doi:10.1590/1678-4685-GMB-2016-0042)
Supplement: Supplementary file 4 [file 1415-4757-gmb-1678-4685-GMB-2016-0042-Suppl09.pdf]

Table S1. ITS1 and ITS2 sequences parameters analyzed per *Passiflora* subgenera: GenBank access, length, lowest energy state, total number of structures, number of hairpins, and percentage of paired bases.

***Astrophea* ITS1**							
**Species**	**GenBank Access**	**Length (Bp)**	**Lowest Engergy State**	**Total number of Structures**	**No. Hairpins**	**No. Paired Nucleotides**	**% No. Paired Nucleotides**
P. amoena	KP769869[a]						
P. arborea	JX470767[b]	268,00	119,90	15,00	7,00	168,00	62,69
P. candida	DQ521279[c]						
P. ceratocarpa	KP769870[a]						
P. citrifolia	AY210939[d]	273,00	117,50	20,00	3,00	168,00	61,54
	AY632707[e]						
P. haematostigma	AY032835[d]	270,00	121,40	17,00	6,00	170,00	62,96
	EU258395 - EU258408[f]						
	EU907230 - EU907234[g]						
P. jussieu	JX470768[b]						
P. kawensis	KP769871[a]						
P. lindeniana	KP769872[a]						
P. macrophylla	AY210944[d]	269,00	118,40	11,00	7,00	164,00	60,97
	DQ458062[h]						
	EU907225 - EU907229[g]						
P. mansoi	AY102361[d]	273,00	121,20	20,00	7,00	172,00	63,00

Astrophea ITS1							
**Species**	**GenBank Access**	**Length (Bp)**	**Lowest Engergy State**	**Total number of Structures**	**No. Hairpins**	**No. Paired Nucleotides**	**% No. Paired Nucleotides**
P. rhamnifolia	KP769873-KP769884[a]						
P. sphaerocarpa	JX470769[b]	268,00	132,50	12,00	5,00	168,00	62,69
P. tina	JX470770[b]						

**Mean**	270,17	121,82	15,83	5,83	168,33	62,31
**Median**	270,00	122,00	16,00	6,00	168,00	62,00
**Max**	273	132,50	20	7	172	63,00
**Min**	268	117,50	11	3	164	60,97

Astrophea ITS2							
**Species**	**GenBank Access**	**Length (Bp)**	**Lowest Engergy State**	**Total number of Structures**	**No. Hairpins**	**No. Paired Nucleotides**	**% No. Paired Nucleotides**
P. amoena	KP769917[a]						
P. arborea	JX470767[b]	204	89,9	13	2	128	62,75
P. candida	DQ521279[c]						
P. ceratocarpa	KP769918[a]						
P. citrifolia	AY210920[d]	222	88,20	15	2	136	61,26
	AY632707[e]						
P. haematostigma	AY032794[d]	212	89,70	16	2	130	61,32
	EU258395 - EU258408[f]						
	EU907230 - EU907234[g]						
P. jussieu	JX470768[b]						
P. kawensis	KP769919[a]						
P. lindeniana	KP769920[a]						
P. macrophylla	AY210925[d]	206	90,60	11	2	124	60,19
	DQ458062[h]						
	EU907225 - EU907230[g]						
P. mansoi	AY102381[d]	207	92,70	13	5	130	62,8

Astrophea ITS2							
**Species**	**GenBank Access**	**Length (Bp)**	**Lowest Engergy State**	**Total number of Structures**	**No. Hairpins**	**No. Paired Nucleotides**	**% No. Paired Nucleotides**
P. rhamnifolia	KP769921-KP769932[a]						
P. sphaerocarpa	JX470769[b]	202	90,60	17	4	124	61,39
P. tina	JX470770[b]						

**Mean**	208,83	90,28	14,17	2,83	128,67	61,62
**Median**	209,00	90,00	14,00	3,00	129,00	62,00
**Max**	222	92,70	17	5	136	62,8
**Min**	202	88,20	11	2	124	60,19

a: Sequences from Giudicelli *et al*.; **b:** Krosnick SE, Porter-Utley KE, MacDougal JM, Jørgensen PM, McDade LA (2013) New insights into the evolution of *Passiflora* subgenus *Decaloba* (Passifloraceae): phylogenetic relationships and morphological synapomorphies. Systematic Botany, 38, 692-713; **c:** Hearn DJ (2006) *Adenia* (Passifloraceae) and its adaptative radiation: Phylogeny and growth form diversification. Systematic Botany, 31, 805-821; **d:** Muschner VC, Lorenz AP, Cervi AC, Bonatto SL, Souza-Chies TT, Salzano FM, Freitas LB (2003). A first molecular phylogenetic analysis of *Passiflora* (Passifloraceae). American Journal of Botany, 90, 1229-1238; **e:** Krosnick SE, Freudenstein JV (2005) Monophyly and floral character homology of old world *Passiflora* (Subgenus *Decaloba*: Supersection *Disemma*). Systematic Botany, 30, 139-152; **f:** Mäder G, Zamberlan PM, Fagundes NJR, Magnus T, Salzano FM, Bonatto SL, Freitas LB (2010) The use and limits of ITS data in the analysis of intraspecific variation in *Passiflora* L. (Passifloraceae). Genetics and Molecular Biology, 33, 99-108; **g:** Mäder G, Magnus T, Lorenz-Lemke AP, et al. ITS subgenera and intraspecific variability in Brazilian *Passiflora*: Understandin molecular evolution. Unpublished; **h:** Krosnick SE, Ford A, Freudenstein JV. Resolving the phylogenetic position of *Hollrungia* and *Tetrapathaea*: The end of two monotypic genera in Passifloraceae. Unpublished.

Decaloba ITS1							
Species	GenBank Access	Length (Bp)	Lowest Engergy State	Total number of Structures	No. Hairpins	No. Paired Nucleotides	% No. Paired Nucleotides
P. anadenia	JX470833[c]	273	90,20	12	7	160	58,61
P. apetala	JX470822[c]	276	113,20	7	7	162	58,7
P. apoda	JX470779[c]	271	93,00	19	7	164	60,52
P. aurantia	DQ521280[d]						
	AY632704[a]						
P. auriculata	AF454804[e]	273	119,80	7	6	178	65,2
	DQ284532[f]						
P. berteroana	JX470780[c]						
P. bicornis	JX470836[c]						
P. bicrura	JX470834[c]						
P. biflora	DQ521281[d]						
	AF454805[e]						
	AY632705[a]						
P. boendery	JX470823[c]						
P. calcicola	JX470813[c]						
P. capsularis	EU258327 - EU258351[g]						
	EU907235 - EU907250[h]						
	AY032837[i]						

Decaloba ITS1							
Species	GenBank Access	Length (Bp)	Lowest Engergy State	Total number of Structures	No. Hairpins	No. Paired Nucleotides	% No. Paired Nucleotides
	JX470806[c]						
P. chelidonea	JX470838[c]						
P. cinnabarina	AY632706[a]	274	112,90	14	6	168	61,31
P. citrina	JX463165[c]						
P. cobanensis	JX470807[c]						
P. cochinchinensis	DQ458080[b]						
	DQ087422						
	AY632714[a]						
P. colimensis	JX470797[c]	269	100,60	20	4	168	62,45
P. complanata	JX470827[c]						
P. coriacea	AF454807[e]						
	AY210940[i]						
	DQ238786[f]						
	JX463147[c]						
	JX470790[c]						
P. cuneata	JX470840[c]						
P. cupiformis	AY632708[a]						
P. cupraea	AY210941[i]						
	JX470815[c]						
P. dolichocarpa	JX470798[c]						
P. eberhardtii	DQ458073[b]						

Decaloba ITS1							
Species	GenBank Access	Length (Bp)	Lowest Engergy State	Total number of Structures	No. Hairpins	No. Paired Nucleotides	% No. Paired Nucleotides
	JX470778[c]						
P. ekmanii	JX470835[c]						
P. escobariana	JX470808[c]	273	88,80	15	5	182	66,67
P. exsudans	JX470799[c]						
P. geminiflora	DQ458076[b]	271	95,30	13	6	150	55,35
P. gracilis	JX470800[c]	273	106,90	15	6	168	61,54
P. guatemalensis	DQ087419[f]	220	84,80	7	4	112	50,91
P. hahnii	JX470777[c]	271	123,80	16	5	170	63,73
P. helleri	AY210942[i]						
	DQ458082[b]						
P. henryi	AY632710[a]						
P. herbertiana	AY632711[a]						
P. hollrungii	DQ458081[b]						
P. holosericea	DQ087417[f]	273	99,70	20	6	172	63
P. inca	JX463163[c]	280	111,70	8	7	174	62,14
P. intricata	JX470844[c]	271	110,20	13	6	176	64,94
P. jianfengensis	DQ458077[b]						
P. jugorum	AY632712[a]						
P. juliana	JX463152 - JX463154[c]						
	JX470791[c]						

Decaloba ITS1							
Species	GenBank Access	Length (Bp)	Lowest Engergy State	Total number of Structures	No. Hairpins	No. Paired Nucleotides	% No. Paired Nucleotides
P. karwinskii	JX470801[c]						
P. kwangtungensis	KF207865[j]						
P. lancearia	JX470845[c]	276	105,20	8	8	172	62,32
P. lancetillensis	AY210943[i]	277	112,60	9	5	170	61,37
	JX470775[c]						
P. lancifolia	JX463158[c]	273	98,90	10	3	170	62,27
	JX470792[c]						
P. leschenaultii	DQ458079[b]						
P. litoralis	JX463107[c]						
	JX463109[c]						
	JX463112 - JX463118[c]						
	JX463123 - JX463126[c]						
	JX463133 - JX463134[c]						
P. lobata	AF454808[e]	274	102,70	19	7	174	63,5
	JX463164[c]						
	JX470802[c]						
P. lobbii	JX463162[c]	276	108,50	12	7	166	60,14

Decaloba ITS1							
Species	GenBank Access	Length (Bp)	Lowest Engergy State	Total number of Structures	No. Hairpins	No. Paired Nucleotides	% No. Paired Nucleotides
P. lobbii subsp. *ayacuchoensis*	JX470782[c]						
P. lutea	DQ006022[k]	275	103,60	10	3	168	61,09
P. membranacea	AY632701[a]	271	129,70	17	5	174	64,21
P. mexicana	AY632713[a]						
P. micropetala	JX470847[c]						
P. microstipula	DQ458066[m]	273	95,80	12	5	162	59,34
P. misera	EU258409 - EU258413[g]						
	AY032838[i]						
P. moluccana var. *glaberrima*	DQ284536[f]						
P. monadelpha	JX470783[c]	272	101,70	18	4	174	63,97
	DQ087418[f]						
P. morifolia	EU258323 - EU258324[g]						
	DQ284533[f]						
P. multiflora	AY210945[i]	272	97,80	12	3	162	59,56
	AY632715						
P. munchiquensis	JX470784[c]	271	109,40	16	3	170	62,73
P. murucuja	AY648559[n]						
	JX470817[c]						

Decaloba ITS1							
**Species**	**GenBank Access**	**Length (Bp)**	**Lowest Engergy State**	**Total number of Structures**	**No. Hairpins**	**No. Paired Nucleotides**	**% No. Paired Nucleotides**
P. oblongata	JX470818[c]						
P. obtusifolia	JX463150 - JX463151[c]						
	JX470793[c]						
P. occidentalis	JX470849[c]						
P. organensis	EU258414 - EU258426[g]						
	AY032839[i]						
P. ornithoura	JX470826[c]						
P. pallida	DQ458084[b]						
	JX463127 - JX463132[c]						
	JX463135 - JX463140[c]						
	JX463142[c]						
P. pardifolia	JX470850[c]						
P. pavonis	JX470831[c]						
P. pedicellaris	JX470776[c]	272	108,80	6	6	172	63,24
P. pendens	JX470803[c]						
P. penduliflora	JX463166[c]						
	JX470820[c]						

Decaloba ITS1							
**Species**	**GenBank Access**	**Length (Bp)**	**Lowest Engergy State**	**Total number of Structures**	**No. Hairpins**	**No. Paired Nucleotides**	**% No. Paired Nucleotides**
P. perakensis	DQ087423[f]						
P. perfoliata	JX463167[c]						
	JX470821[c]						
P. pilosa	JX470804[c]						
P. podlechii	JX463161[c]	276	111,10	10	7	172	62,32
P. pohlii	EU258325[g]	275	108,60	11	5	166	60,36
P. punctata	AY210946[i]						
P. pusilla	JX470809[c]						
P. rovirosae	JX470810[c]	272	95,20	8	7	166	61,03
P. rubra	AY032836[i]						
	JX470811[c]						
P. rufa	AY210948[i]	272	102,90	5	6	168	61,76
P. rugosissima	JX470828[c]	271	101,90	12	7	170	62,73
P. sagasteguii	JX470785[c]	277	107,10	16	7	166	59,93
P. sandrae	JX470852[c]						
P. sanguinolenta	JX470812[c]						
P. sexflora	AY210949[i]						
	JX463168[c]						
	JX470829 - JX470830[c]						

Decaloba ITS1							
**Species**	**GenBank Access**	**Length (Bp)**	**Lowest Engergy State**	**Total number of Structures**	**No. Hairpins**	**No. Paired Nucleotides**	**% No. Paired Nucleotides**
P. sexocellata	JX463143 - JX463146[c]						
P. siamica	DQ458212 - DQ458216[b]						
	DQ087424[f]						
	AY632717[a]						
P. sodiroi	JX470786[c]	271	100,80	16	3	168	61,99
P. solomonii	JX470787[c]	271	101,70	16	7	166	61,25
P. suberosa	AY032841[i]						
	AF454806[e]						
	AY632718[a]						
P. suberosa var. *suberosa*	JX463108[c]						
	JX463110[c]						
	JX463119 - JX463122[c]						
P. tacanensis	JX470794[c]	271	96,60	12	6	168	61,99
P. tatei	JX470853[c]						
P. telesiphe	JX470854[c]						
P. tenella	JX470832[c]	273	99,60	13	4	176	64,47

Decaloba ITS1							
**Species**	**GenBank Access**	**Length (Bp)**	**Lowest Engergy State**	**Total number of Structures**	**No. Hairpins**	**No. Paired Nucleotides**	**% No. Paired Nucleotides**
P. tenuiloba	JX463159 - JX463160[c]	272	93,30	12	6	166	61,03
	AY632719[a]						
P. tonkinensis	DQ087425[f]						
P. tricuspis	EU258455 - EU258460[g]						
	AY102348[i]						
	JX470855[c]						
P. trifasciata	KP769885[l]						
P. truncata	AY102354[i]	272	114,80	9	7	174	63,97
	JX470788[c]						
P. tuberosa	JX470856[c]						
P. urnifolia	EU258461 - EU258465[g]						
	JX470857[c]						
P. cf. *viridescens*	JX470859[c]						
P. viridiflora	JX463155 - JX463156[c]						
P. wilsonii	DQ458072[b]						
	DQ087426						
P. xiizkodz	AY210950[i]						

Decaloba ITS1							
**Species**	**GenBank Access**	**Length (Bp)**	**Lowest Engergy State**	**Total number of Structures**	**No. Hairpins**	**No. Paired Nucleotides**	**% No. Paired Nucleotides**
	DQ238786[o]						
	JX463103 - JX463104[c]						
	JX463106[c]						
	JX470795[c]						
P. xiizkodz subsp. i*tzensis*	JX463101[c]						
P. xishuangbannaensis	DQ458071[b]	278	103,60	17	4	178	64,03

**Mean**	271,82	104,17	12,62	5,56	167,74	61,68
**Median**	272	104	13	6	168	62
**Max**	280	129,7	20	8	182	66,67
**Min**	220	84,8	5	3	112	50,91

Decaloba ITS2							
**Species**	**GenBank Access**	**Length (Bp)**	**Lowest Engergy State**	**Total number of Structures**	**No. Hairpins**	**No. Paired Nucleotides**	**% No. Paired Nucleotides**
P. anadenia	JX470833[c]	208	73,20	6	3	128	61,54
P. apetala	JX470822[c]	206	77,40	11	3	130	63,11
P. apoda	JX470779[c]	208	73,70	12	4	116	55,77
P. aurantia	DQ521280[d]						
	AY632704[a]						
P. auriculata	AF454804[e]	209	75,00	7	4	118	56,46
	DQ284532[f]						
P. berteroana	JX470780[c]						
P. bicornis	JX470836[c]						
P. bicrura	JX470834[c]						
P. biflora	DQ521281[d]						
	AF454805[e]						
	AY632705[a]						
P. boendery	JX470823[c]						
P. calcicola	JX470813[c]						
P. capsularis	EU258327 - EU258351[g]						
	EU907235 - EU907250[h]						

Decaloba ITS2							
**Species**	**GenBank Access**	**Length (Bp)**	**Lowest Engergy State**	**Total number of Structures**	**No. Hairpins**	**No. Paired Nucleotides**	**% No. Paired Nucleotides**
	AY032796[i]						
	JX470806[c]						
P. chelidonea	JX470838[c]						
P. cinnabarina	AY632706[a]	208	74,90	9	3	120	57,69
P. citrina	JX463165[c]						
P. cobanensis	JX470807[c]						
P. cochinchinensis	DQ458080[b]						
	DQ087422						
	AY632714[a]						
P. colimensis	JX470797[c]	208	73,30	9	3	124	59,62
P. complanata	JX470827[c]						
P. coriacea	AF454807[e]						
	AY210921[i]						
	DQ238786[f]						
	JX463147[c]						
	JX470790[c]						
P. cuneata	JX470840[c]						
P. cupiformis	AY632708[a]						
P. cupraea	AY210922[i]						
	JX470815[c]						
P. dolichocarpa	JX470798[c]						

Decaloba ITS2							
**Species**	**GenBank Access**	**Length (Bp)**	**Lowest Engergy State**	**Total number of Structures**	**No. Hairpins**	**No. Paired Nucleotides**	**% No. Paired Nucleotides**
P. eberhardtii	DQ458073[b]						
	JX470778[c]						
P. ekmanii	JX470835[c]						
P. escobariana	JX470808[c]	211	75,20	7	4	134	63,51
P. exsudans	JX470799[c]						
P. geminiflora	DQ458076[b]	210	75,80	11	3	120	57,14
P. gracilis	JX470800[c]	213	86,30	6	4	136	63,85
P. guatemalensis	DQ087419[f]	203	89,00	17	4	130	64,04
P. hahnii	JX470777[c]	209	85,20	14	3	130	62,2
P. helleri	AY210923[i]						
	DQ458082[b]						
P. henryi	AY632710[a]						
P. herbertiana	AY632711[a]						
P. hollrungii	DQ458081[b]						
P. holosericea	DQ087417[f]	211	77,10	8	3	132	62,56
P. inca	JX463163[c]	209	76,00	12	3	124	59,33
P. intricata	JX470844[c]	208	74,60	6	3	124	59,62
P. jianfengensis	DQ458077[b]						
P. jugorum	AY632712[a]						
P. juliana	JX463152 - JX463154[c]						

Decaloba ITS2							
**Species**	**GenBank Access**	**Length (Bp)**	**Lowest Engergy State**	**Total number of Structures**	**No. Hairpins**	**No. Paired Nucleotides**	**% No. Paired Nucleotides**
	JX470791[c]						
P. karwinskii	JX470801[c]						
P. kwangtungensis	KF207865[j]						
P. lancearia	JX470845[c]	209	74,30	6	3	122	58,37
P. lancetillensis	AY210924[i]	199	66,70	12	4	128	64,32
	JX470775[c]						
P. lancifolia	JX463158[c]	208	66,40	9	3	116	55,77
	JX470792[c]						
P. leschenaultii	DQ458079[b]						
P. litoralis	JX463107[c]						
	JX463109[c]						
	JX463112 - JX463118[c]						
	JX463123 - JX463126[c]						
	JX463133 - JX463134[c]						
P. lobata	AF454808[e]	207	70,00	10	3	118	57
	JX463164[c]						
	JX470802[c]						
P. lobbii	JX463162[c]	209	75,00	14	3	122	58,37

Decaloba ITS2							
**Species**	**GenBank Access**	**Length (Bp)**	**Lowest Engergy State**	**Total number of Structures**	**No. Hairpins**	**No. Paired Nucleotides**	**% No. Paired Nucleotides**
P. lobbii subsp. *ayacuchoensis*	JX470782[c]						
P. lutea	DQ006022[k]	209	89,10	5	3	128	61,24
P. membranacea	AY632701[a]	210	84,30	16	3	124	59,05
P. mexicana	AY632713[a]						
P. micropetala	JX470847[c]						
P. microstipula	DQ458066[m]	210	79,50	9	5	132	62,86
P. misera	EU258409 - EU258413[g]						
	AY032797[i]						
P. moluccana var. *glaberrima*	DQ284536[f]						
P. monadelpha	JX470783[c]	208	77,20	6	4	120	57,69
	DQ087418[f]						
P. morifolia	EU258323 - EU258324[g]						
	DQ284533[f]						
P. multiflora	AY210926[i]	202	68,70	16	3	120	59,41
	AY632715						
P. munchiquensis	JX470784[c]	208	72,10	12	4	114	54,81
P. murucuja	AY648559[n]						

Decaloba ITS2							
**Species**	**GenBank Access**	**Length (Bp)**	**Lowest Engergy State**	**Total number of Structures**	**No. Hairpins**	**No. Paired Nucleotides**	**% No. Paired Nucleotides**
	JX470817[c]						
P. oblongata	JX470818[c]						
P. obtusifolia	JX463150 - JX463151[c]						
	JX470793[c]						
P. occidentalis	JX470849[c]						
P. organensis	EU258414 - EU258426[g]						
	AY032798[i]						
P. ornithoura	JX470826[c]						
P. pallida	DQ458084[b]						
	JX463127 - JX463132[c]						
	JX463135 - JX463140[c]						
	JX463142[c]						
P. pardifolia	JX470850[c]						
P. pavonis	JX470831[c]						
P. pedicellaris	JX470776[c]	216	85,70	20	3	130	60,19
P. pendens	JX470803[c]						
P. penduliflora	JX463166[c]						

Decaloba ITS2							
**Species**	**GenBank Access**	**Length (Bp)**	**Lowest Engergy State**	**Total number of Structures**	**No. Hairpins**	**No. Paired Nucleotides**	**% No. Paired Nucleotides**
	JX470820[c]						
P. perakensis	DQ087423[f]						
P. perfoliata	JX463167[c]						
	JX470821[c]						
P. pilosa	JX470804[c]						
P. podlechii	JX463161[c]	209	75,00	14	3	122	58,37
P. pohlii	EU258325[g]	201	72,90	8	4	130	64,68
P. punctata	AY210927[i]						
P. pusilla	JX470809[c]						
P. rovirosae	JX470810[c]	211	64,30	12	4	120	56,87
P. rubra	AY032795[i]						
	JX470811[c]						
P. rufa	AY210929[i]	203	70,50	13	3	116	57,14
P. rugosissima	JX470828[c]	208	71,40	10	2	118	56,73
P. sagasteguii	JX470785[c]	209	74,00	13	3	124	59,33
P. sandrae	JX470852[c]						
P. sanguinolenta	JX470812[c]						
P. sexflora	AY210930[i]						
	JX463168[c]						
	JX470829 - JX470830[c]						

Decaloba ITS2							
**Species**	**GenBank Access**	**Length (Bp)**	**Lowest Engergy State**	**Total number of Structures**	**No. Hairpins**	**No. Paired Nucleotides**	**% No. Paired Nucleotides**
P. sexocellata	JX463143 - JX463146[c]						
P. siamica	DQ458212 - DQ458216[b]						
	DQ087424[f]						
	AY632717[a]						
P. sodiroi	JX470786[c]	208	69,20	20	3	124	59,62
P. solomonii	JX470787[c]	209	76,20	14	3	124	59,33
P. suberosa	AY032800[i]						
	AF454806[e]						
	AY632718[a]						
P. suberosa var. *suberosa*	JX463108[c]						
	JX463110[c]						
	JX463119 - JX463122[c]						
P. tacanensis	JX470794[c]	208	65,80	14	2	126	60,58
P. tatei	JX470853[c]						
P. telesiphe	JX470854[c]						
P. tenella	JX470832[c]	212	70,40	17	2	108	50,94

Decaloba ITS2							
**Species**	**GenBank Access**	**Length (Bp)**	**Lowest Engergy State**	**Total number of Structures**	**No. Hairpins**	**No. Paired Nucleotides**	**% No. Paired Nucleotides**
P. tenuiloba	JX463159 - JX463160[c]	208	65,20	15	3	120	57,69
	AY632719[a]						
P. tonkinensis	DQ087425[f]						
P. tricuspis	EU258455 - EU258460[g]						
	AY102368[i]						
	JX470855[c]						
P. trifasciata	KP769933[l]						
P. truncata	AY102374[i]	205	76,10	8	4	116	56,59
	JX470788[c]						
P. tuberosa	JX470856[c]						
P. urnifolia	EU258461 - EU258465[g]						
	JX470857[c]						
P. cf. *viridescens*	JX470859[c]						
P. viridiflora	JX463155 - JX463156[c]						
P. wilsonii	DQ458072[b]						
	DQ087426						
P. xiizkodz	AY210931[i]						

Decaloba ITS2							
Species	GenBank Access	Length (Bp)	Lowest Engergy State	Total number of Structures	No. Hairpins	No. Paired Nucleotides	% No. Paired Nucleotides
	DQ238786[o]						
	JX463103 - JX463104[c]						
	JX463106[c]						
	JX470795[c]						
P. xiizkodz subsp. i*tzensis*	JX463101[c]						
P. xishuangbannaensis	DQ458071[b]	208	75,20	8	3	124	59,62

**Mean**	208,08	74,92	11,18	3,26	123,38	59,31
**Median**	208	75,00	11	3	123	59,00
**Max**	216	89,10	20	5	136	64,68
**Min**	199	64,30	5	2	108	50,94

a: Krosnick SE, Freudenstein JV (2005) Monophyly and floral character homology of old world *Passiflora* (Subgenus *Decaloba*: Supersection *Disemma*). Systematic Botany, 30, 139-152; **b:** Krosnick SE, Freudenstein JV. Phylogenetic relationships among the Old World species of *Passiflora* L. (Subgenus *Decaloba*: Supersection *Disemma*). Unpublished; **c:** Krosnick SE, Porter-Utley KE, MacDougal JM, Jørgensen PM, McDade LA (2013) New insights into the evolution of *Passiflora* subgenus *Decaloba* (Passifloraceae): phylogenetic relationships and morphological synapomorphies. Systematic Botany, 38, 692-713; **d:** Hearn DJ (2006) *Adenia* (Passifloraceae) and its adaptative radiation: Phylogeny and growth form diversification. Systematic Botany, 31, 805-821; **e:** Ossowski AM, Hunter FF. Coevolution of *Heliconius* spp. and *Passiflora* spp.: A phylogenetic comparison. Unpublished; **f:** Krosnick SE, Freudenstein JV (2006) Patterns of anomalous floral development in the Asian *Passiflora* (subgenus *Decaloba*: supersection *Disemma*). Am. J. Bot., 93, 620-636; **g:** Mäder G, Zamberlan PM, Fagundes NJR, Magnus T, Salzano FM, Bonatto SL, Freitas LB (2010) The use and limits of ITS data in the analysis of intraspecific variation in *Passiflora* L. (Passifloraceae).

Decaloba ITS2							
**Species**	**GenBank Access**	**Length (Bp)**	**Lowest Engergy State**	**Total number of Structures**	**No. Hairpins**	**No. Paired Nucleotides**	**% No. Paired Nucleotides**

Genetics and Molecular Biology, 33, 99-108; **h:** Mäder G, Magnus T, Lorenz-Lemke AP, et al. ITS subgenera and intraspecific variability in Brazilian *Passiflora*: Understandin molecular evolution. Unpublished; **i:** Muschner VC, Lorenz AP, Cervi AC, Bonatto SL, Souza-Chies TT, Salzano FM, Freitas LB (2003). A first molecular phylogenetic analysis of *Passiflora* (Passifloraceae). American Journal of Botany, 90, 1229-1238; **j:** Krosnick SE, Xun-Lin Y, Deng Y (2013) The rediscovery of *Passiflora kwangtungensis* Merr. (subgenus *Decaloba* supersection *Disemma*): a critically endangered Chinese endemic. PhytoKeys, 23, 55-74; **k:** Kress WJ, Wurdack KJ, Zimmer EA, Weigt LA, Janzen DH (2005). Use of DNA barcodes to identify flowering plants. Proc. Natl. Acad. Sci. U.S.A. 102, 8369-8374; **l:** Sequences from Giudicelli *et al*.; **m:** Krosnick SE, Ford A, Freudenstein JV. Resolving the phylogenetic position of *Hollrungia* and *Tetrapathaea*: The end of two monotypic genera in Passifloraceae. Unpublished; **n:** Kay EE. Floral Evolutionary Ecology of *Passiflora*: subgenera *Murucuia*, *Pseudomurucuja* and *Astephia*. Unpublished; **o:** Muschner VC, Lorenz-Lemke AP, Vecchia M, Bonatto SL, Salzano FM, Freitas LB. Differential organellar inheritance in *Passiflora* (Passifloraceae) subgenera. Unpublished.

Deidamioides ITS1							
Species	GenBank Access	Length (Bp)	Lowest Engergy State	Total number of Structures	No. Hairpins	No. Paired Nucleotides	% No. Paired Nucleotides
P. arbelaezii	DQ521278[a]	272	108,80	20	4	164	60,29
	AY632703[b]						
P. contracta	KF196619 - KF196691[d]						
	KF196635[d]	232	108,40	10	4	138	59,48
P. deidamioides	EU907257 - EU907265[e]	235	99,40	20	2	138	58,72
P. discophora	DQ458061[c]						
	JX470772[f]	266	106,10	19	7	158	59,4
P. gracillima	JX470773[f]	269	106,40	11	7	160	59,48
P. ovalis	KF196601 - KF196618[d]						
	KF196606[d]	233	97,10	16	7	138	59,23

**Mean**	215,29	89,46	13,71	4,43	128,00	50,94
**Median**	245,00	101,00	15,00	5,00	145,00	59,00
**Max**	272	108,80	20	7	164	60,29
**Min**	205	81,10	10	2	116	56,59

Deidamioides ITS2							
Species	GenBank Access	Length (Bp)	Lowest Engergy State	Total number of Structures	No. Hairpins	No. Paired Nucleotides	% No. Paired Nucleotides
P. arbelaezii	DQ521278[a]	200	83,30	13	2	134	67,00
	AY632703[b]						
P. contracta	KF196619 - KF196691[d]						
	KF196635[d]	210	98,30	9	2	130	61,90
P. deidamioides	EU907257 - EU907265[e]	209	89,10	13	2	124	59,33
P. discophora	DQ458061[c]						
	JX470772[f]	200	81,10	17	4	128	64,00
P. gracillima	JX470773[f]	200	79,60	19	2	124	62,00
P. ovalis	KF196601 - KF196618[d]						
	KF196606[d]	208	95,80	12	2	132	63,46

**Mean**	175,29	75,31	11,86	2,00	110,29	53,96
**Median**	206,00	89,00	15,00	2,00	130,00	63,00
**Max**	212	98,30	19	4	138	67,00
**Min**	200	79,60	9	2	124	59,33

a: Hearn DJ (2006) *Adenia* (Passifloraceae) and its adaptative radiation: Phylogeny and growth form diversification. Systematic Botany, 31, 805-821; **b:** Krosnick SE, Freudenstein JV (2005) Monophyly and floral character homology of old world *Passiflora* (Subgenus *Decaloba*: Supersection *Disemma*). Systematic Botany, 30, 139-152; **c:** Krosnick SE, Ford A, Freudenstein JV. Resolving the phylogenetic position of *Hollrungia* and *Tetrapathaea*: The end of two monotypic genera in Passifloraceae. Unpublished; **d:** Cazé ALR, Mäder G, Bonatto SL, Freitas LB (2013) A molecular systematic analysis of *Passiflora ovalis* and *Passiflora contracta* (Passifloraceae). Phytotaxa, 132, 39-46; **e:** Mäder G, Magnus T, Lorenz-Lemke AP, et al. ITS subgenera and intraspecific variability in Brazilian *Passiflora*: Understandin molecular evolution. Unpublished; **f:** Krosnick SE, Porter-Utley KE, MacDougal JM, Jørgensen PM, McDade LA (2013) New insights into the evolution of *Passiflora* subgenus *Decaloba* (Passifloraceae): phylogenetic relationships and morphological synapomorphies. Systematic Botany, 38, 692-713.

Passiflora ITS1							
**Species**	**GenBank Access**	**Length (Bp)**	**Lowest Engergy State**	**Total number of Structures**	**No. Hairpins**	**No. Paired Nucleotides**	**% No. Paired Nucleotides**
P. actinia	AY032832[a]	226	109,20	5	4	142	62,83
	AY542629 - AY542644[b]						
	AY219240 - AY219255[b]						
P. acuminata	KP769886[c]						
P. alata	AY032826[a]	225	92,40	9	4	124	55,11
	AY858145 - AY858229[d]						
	AF454800[e]						
P. amethystina	AY102347[a]	228	106,20	5	4	124	54,39
	EU258307 - EU258309[f]						
P. ampullacea	AY632720[g]						
P. caerulea	EU258310 - EU258316[f]						
	AY032824[a]						
	AF454802[e]						
P. campanulata	AY032829[a]						
P. cerasina	KP769887[c]						

Passiflora ITS1							
Species	GenBank Access	Length (Bp)	Lowest Engergy State	Total number of Structures	No. Hairpins	No. Paired Nucleotides	% No. Paired Nucleotides
P. cincinnata	EU258353 - EU258358[f]						
	DQ344629[h]						
	AY102363[a]						
P. coccinea	KP769888[c]						
P. edmundoi	EU258370[f]						
	EU258373 - EU258374[f]						
	AY102351[a]						
P. edulis	EU258375 - EU258384[f]						
	AY032831[a]						
	JX470774[i]						
	AF454803[e]						
P. eichleriana	EU258317 - EU258319[f]						
	AY102346[a]						
P. elegans	AY032833[a]						
	AY542645 - AY542657[b]						

Passiflora ITS1							
**Species**	**GenBank Access**	**Length (Bp)**	**Lowest Engergy State**	**Total number of Structures**	**No. Hairpins**	**No. Paired Nucleotides**	**% No. Paired Nucleotides**
	AY219256 - AY219262[b]						
P. foetida	AY032834[a]	229	102,00	6	5	144	62,88
	EU258385 - EU258390[f]						
	EU258393 - EU258394[f]						
	DQ521376[j]						
	DQ238783[h]						
	DQ458053[k]						
	DQ499117[l]						
	JQ723359[m]						
P. gabrielliana	AY210953[a]						
P. galbana	AY032843[a]						
P. garckei	AY210952[a]						
P. hatsbachii	KP769889[c]						
P. ishnoclada	KP769890[c]						
P. jervensis	KP769891[c]						
P. jilekii	AY102360[a]	225	105,00	7	4	138	61,33
	EU258320 - EU258321[f]						

Passiflora ITS1							
Species	GenBank Access	Length (Bp)	Lowest Engergy State	Total number of Structures	No. Hairpins	No. Paired Nucleotides	% No. Paired Nucleotides
P. kermesina	AY032825[a]						
P. laurifolia	KP769892[c]						
P. loefgrenii	KP769893[c]						
P. luetzelburgii	KP769894[c]						
P. maliformis	AY210956[a]						
P. mathewsii	KP769895[c]	225	108,20	12	5	138	61,33
P. mendoncaei	AY102358[a]						
P. miersii	EU258322[f]						
	EU907266 - EU907269[n]						
	AY102350[a]						
P. mixta	KP769896[c]						
P. mucronata	AY210951[a]						
P. mucugensis	KP769897[c]						
P. nitida	KP769898[c]						
P. odontophylla	KP769899[c]						
P. oerstedii	AF454797[e]						
P. palmeri	DQ238784[h]						
P. pilosicorona	KP769900[c]						
P. platyloba	AF454798[e]						
P. quadrangularis	AY032827[a]						

Passiflora ITS1							
**Species**	**GenBank Access**	**Length (Bp)**	**Lowest Engergy State**	**Total number of Structures**	**No. Hairpins**	**No. Paired Nucleotides**	**% No. Paired Nucleotides**
	AY636107[g]						
	AF454799[e]						
P. racemosa	KP769901[c]	222	93,20	9	4	132	59,46
P. recurva	AY102349[a]						
P. serratifolia	AY210954[a]						
P. serratodigitata	AY210957[a]	229	101,50	17	4	140	61,14
	AY636108[g]						
P. setacea	AY102356[a]						
P. setulosa	AY032828[a]	223	98,50	9	2	136	60,99
P. sidiifolia	EU258435 - EU258445[f]						
	AY102353[a]						
P. speciosa	AY102362[a]						
P. sprucei	KP769902[c]						
P. tenuifila	EU258446 - EU258454[f]						
P. trifoliata	KP769903[c]						
P. tripartita	KP769904[c]						
P. trisecta	KP769905[c]						
P. urubiciensis	EU258326[f]						
	AY102355[a]						

Passiflora ITS1							
Species	GenBank Access	Length (Bp)	Lowest Engergy State	Total number of Structures	No. Hairpins	No. Paired Nucleotides	% No. Paired Nucleotides
P. villosa	EU258391 - EU258392[f]						
	EU258466 - EU258469[f]						
	AY102357[a]						
P. vitifolia	AF454796[e]						

**Mean**	225,78	101,80	8,78	4,00	135,33	59,94
**Median**	226	102	9	4	135	60
**Max**	229	109,20	17	5	144	62,88
**Min**	222	92,40	5	2	124	54,39

Passiflora ITS2							
**Species**	**GenBank Access**	**Length (Bp)**	**Lowest Engergy State**	**Total number of Structures**	**No. Hairpins**	**No. Paired Nucleotides**	**% No. Paired Nucleotides**
P. actinia	AY032791[a]	176	79,40	12	3	108	61,36
	AY219264 - AY219279[b]						
	AY542658 - AY542673[b]						
P. acuminata	KP769934[c]						
P. alata	AY032785[a]	202	91,40	20	2	140	69,31
	AY858263 - AY858347[d]						
	AF454800[e]						
P. amethystina	AY102367[a]	204	100,70	20	5	138	67,65
	EU258307 - EU258309[f]						
P. ampullacea	AY632720[g]						
P. caerule\a	EU258310 - EU258316[f]						
	AY032782[a]						
	AF454802[e]						
P. campanulata	AY032788[a]						
P. cerasina	KP769935[c]						

Passiflora ITS2							
Species	GenBank Access	Length (Bp)	Lowest Engergy State	Total number of Structures	No. Hairpins	No. Paired Nucleotides	% No. Paired Nucleotides
P. cincinnata	EU258353 - EU258358[f]						
	DQ344629[h]						
	AY102383[a]						
P. coccinea	KP769936[c]						
P. edmundoi	EU258370[f]						
	EU258373 - EU258374[f]						
	AY102371[a]						
P. edulis	EU258375 - EU258384[f]						
	AY032790[a]						
	JX470774[i]						
	AF454803[e]						
P. eichleriana	EU258317 - EU258319[f]						
	AY102366[a]						
P. elegans	AY032792[a]						
	AY219280 - AY219286[b]						

Passiflora ITS2							
Species	GenBank Access	Length (Bp)	Lowest Engergy State	Total number of Structures	No. Hairpins	No. Paired Nucleotides	% No. Paired Nucleotides
	AY542674 - AY542686[b]						
P. foetida	AY032793[a]	187	92,50	14	2	124	66,31
	EU258385 - EU258390[f]						
	EU258393 - EU258394[f]						
	DQ521376[j]						
	DQ238783[h]						
	DQ458053[k]						
	DQ499117[l]						
	JQ723359[m]						
P. gabrielliana	AY210934[a]						
P. galbana	AY032784[a]						
P. garckei	AY210933[a]						
P. hatsbachii	KP769937[c]						
P. ishnoclada	KP769938[c]						
P. jervensis	KP769939[c]						
P. jilekii	AY102380[a]	203	95,70	20	3	132	65,02
	EU258320 - EU258321[f]						

Passiflora ITS2							
**Species**	**GenBank Access**	**Length (Bp)**	**Lowest Engergy State**	**Total number of Structures**	**No. Hairpins**	**No. Paired Nucleotides**	**% No. Paired Nucleotides**
P. kermesina	AY032783[a]						
P. laurifolia	KP769940[c]						
P. loefgrenii	KP769941[c]						
P. luetzelburgii	KP769942[c]						
P. maliformis	AY210937[a]						
P. mathewsii	KP769943[c]	181	89,30	20	3	124	68,51
P. mendoncaei	AY102378[a]						
P. miersii	EU258322[f]						
	EU907266 - EU907269[n]						
	AY102370[a]						
P. mixta	KP769944[c]						
P. mucronata	AY210932[a]						
P. mucugensis	KP769945[c]						
P. nitida	KP769946[c]						
P. odontophylla	KP769947[c]						
P. oerstedii	AF454797[e]						
P. palmeri	DQ238784[h]						
P. pilosicorona	KP769948[c]						
P. platyloba	AF454798[e]						
P. quadrangularis	AY032786[a]						

Passiflora ITS2							
**Species**	**GenBank Access**	**Length (Bp)**	**Lowest Engergy State**	**Total number of Structures**	**No. Hairpins**	**No. Paired Nucleotides**	**% No. Paired Nucleotides**
	AY636107[g]						
	AF454799[e]						
P. racemosa	KP769949[c]	202	98,20	11	2	140	69,31
P. recurva	AY102369[a]						
P. serratifolia	AY210935[a]						
P. serratodigitata	AY210938[a]	202	94,10	19	3	138	68,32
	AY636108[g]						
P. setacea	AY102376[a]						
P. setulosa	AY032787[a]	201	94,60	20	2	132	65,67
P. sidiifolia	EU258435 - EU258445[f]						
	AY102373[a]						
P. speciosa	AY102382[a]						
P. sprucei	KP769950[c]						
P. tenuifila	EU258446 - EU258454[f]						
P. trifoliata	KP769951[c]						
P. tripartita	KP769952[c]						
P. trisecta	KP769953[c]						
P. urubiciensis	EU258326[f]						
	AY102375[a]						

Passiflora ITS2							
Species	GenBank Access	Length (Bp)	Lowest Engergy State	Total number of Structures	No. Hairpins	No. Paired Nucleotides	% No. Paired Nucleotides
P. villosa	EU258391 - EU258392[f]						
	EU258466 - EU258469[f]						
	AY102377[a]						
P. vitifolia	AF454796[e]						

**Mean**	195,33	92,88	17,33	2,78	130,67	66,83
**Median**	195	93	17	3	131	67
**Max**	204	100,70	20	5	140	69,31
**Min**	176	79,40	11	2	108	61,36

a: Muschner VC, Lorenz AP, Cervi AC, Bonatto SL, Souza-Chies TT, Salzano FM, Freitas LB (2003). A first molecular phylogenetic analysis of *Passiflora* (Passifloraceae). American Journal of Botany, 90, 1229-1238; **b:** Lorenz-Lemke AP, Muschner VC, Bonatto SL, Cervi AC, Salzano FM, Freitas LB (2005) Phylogeographic inferences concerning evolution of Brazilian *Passiflora actinia* and *P. elegans* (Passifloraceae) based on ITS (nrDNA) variation. Annals of Botany, 95, 799-806; **c:** Sequences from Giudicelli *et al.*; **d:** Koehler-Santos P, Lorenz-Lemke AP, Muschner VC, Salzano FM, Freitas LB. Evolutionary implications of the intrapopulation diversity of *Passiflora alata.* Unpublished; **e:** Ossowski AM, Hunter FF. Coevolution of *Heliconius* spp. and *Passiflora* spp.: A phylogenetic comparison. Unpublished; **f:** Mäder G, Zamberlan PM, Fagundes NJR, Magnus T, Salzano FM, Bonatto SL, Freitas LB (2010) The use and limits of ITS data in the analysis of intraspecific variation in *Passiflora* L. (Passifloraceae). Genetics and Molecular Biology, 33, 99-108; **g:** Krosnick SE, Freudenstein JV (2005) Monophyly and floral character homology of old world *Passiflora* (Subgenus *Decaloba*: Supersection *Disemma*). Systematic Botany, 30, 139-152; **h:** Muschner VC, Lorenz-Lemke AP, Vecchia M, Bonatto SL, Salzano FM, Freitas LB. Differential organellar inheritance in *Passiflora* (Passifloraceae) subgenera. Unpublished; **i:** Krosnick SE, Porter-Utley KE, MacDougal JM, Jørgensen PM, McDade LA (2013) New insights into the evolution of *Passiflora* subgenus *Decaloba* (Passifloraceae): phylogenetic relationships and morphological synapomorphies. Systematic Botany, 38, 692-713; **j:** Hearn DJ (2006) *Adenia* (Passifloraceae) and its adaptative radiation: Phylogeny and growth form diversification. Systematic Botany, 31, 805-821; **k:** Krosnick SE, Ford A, Freudenstein JV. Resolving the phylogenetic position of *Hollrungia* and *Tetrapathaea*: The end of two monotypic genera in Passifloraceae. Unpublished; **l:** Wright S, Keeling J, Gillman L (2006) The road from santa Rosalia: a faster tempo of evolution on tropical climes. Proceedings of the National Academy of Sciences of the United States of America, 103, 7718-7722; **m:** Thulin M, Razafimandimbison SG, Chafe P, Heidari N, Kool A, Shore JS (2012) Phyloheny of the Turneracea clade (Passifloraceae): Trans-Atlantic disjunctions and two new genera in Africa. Taxon, 61, 308-323; **n:** Mäder G, Magnus T, Lorenz-Lemke AP, et al. ITS subgenera and intraspecific variability in Brazilian *Passiflora*: Understandin molecular evolution. Unpublished.
