# Supplementary material for: Secondary structure of nrDNA Internal Transcribed Spacers as a useful tool to align highly divergent species in phylogenetic studies
Source: Genet Mol Biol. 2017 Feb 13;40(1 Suppl 1):191–9. doi: 10.1590/1678-4685-GMB-2016-0042 (PMC5452138; doi:10.1590/1678-4685-GMB-2016-0042)
Supplement: Supplementary file 2 [file 1415-4757-gmb-1678-4685-GMB-2016-0042-Suppl08.pdf]

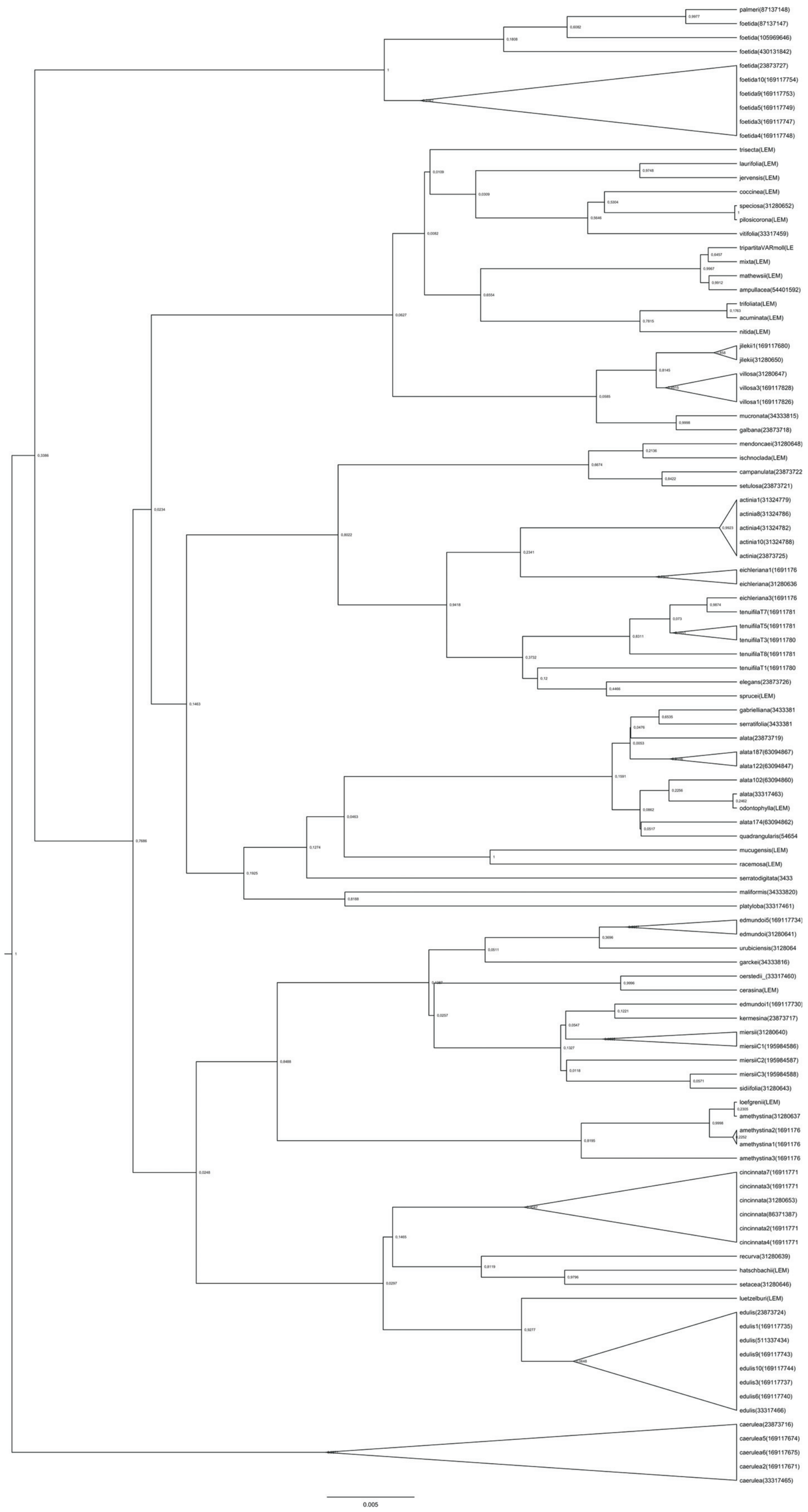

Figure S8 - Phylogenetic trees based on ITS sequences for *Passiflora*, ITS2.
